# Optimization of Microwave-Assisted Pectin Extraction from Cocoa Pod Husk

**DOI:** 10.3390/molecules27196544

**Published:** 2022-10-03

**Authors:** Maya Sarah, Isti Madinah Hasibuan, Erni Misran, Seri Maulina

**Affiliations:** Department of Chemical Engineering, Universitas Sumatera Utara, Kota Medan 20222, Indonesia

**Keywords:** pectin, extraction, cocoa pod husk, microwave, optimization

## Abstract

Optimization of pectin extraction from cocoa pod husk was carried out to examine independent variables that affect the quality of pectin obtained and determine the best conditions for the extraction process. In this study, three independent variables thought to contribute to microwave-assisted pectin extraction were used, namely, extraction time (20, 25, and 30 min), microwave power (180, 300, and 450 W), and citric acid concentration (2.5%, 5%, and 10%). Response surface methodology was applied using central composite design (CCD) to examine the effect of independent variables on the parameter measured. Among the seven parameters measured, namely, yield, temperature, moisture content, ash content, equivalent weight, methoxyl content, and galacturonic acid content, the statistical analysis test showed that the model equations that were suitable for predicting response values were temperature, moisture content, and ash content. Therefore, the optimization of conditions was only reviewed for these three parameters. Optimization results concluded that the factors that had the most significant effect on temperature, moisture content, and ash content were extraction time and microwave power. Optimal conditions for pectin extraction were at 30 min extraction time, 450 W microwave power, and 10% solvent concentration, resulting in a temperature of 104 °C, moisture content of 6%, and ash content of 8%. Other parameter values measured under these conditions were a yield of 21.10%, equivalent weight of 602.40 mg/mol, methoxyl content of 6.07%, and galacturonic acid content of 72.86%. Pectin extracted from cocoa pod husk using this method has the potential to be further applied, especially in the food and pharmaceutical industries.

## 1. Introduction

Pectin is a structural polysaccharide in plant cell walls. The main unit is galacturonic acid with various compositions, structures, and molecular weight. Pectin has long been used as an ingredient in the food and beverage industry. Its main function is as a thickening, stabilizing, emulsifying, and gelling agent. In addition to the food industry, pectin is useful in the medical field as a drug carrier such as for nasal, eye, and oral drug delivery [1]. Pectin is used in the pharmaceutical industry as a natural polymer for drug delivery to the gastrointestinal tract, such as matrix tablets, gel beads, and film-coated dosage forms. Natural pectin benefits are considered better by scientists and consumers because of its biodegradability [2].

Agro-industrial waste must be transformed into value-added products to meet the demand created by the expanding population. The conversion of natural polymers has increased tremendously because of their easy process to become value-added products. Pectin is one of the natural polymers that stands out because it is applied to a variety of pharmaceutical and therapeutic applications [3]. The use of various natural resources and extraction methods to produce pectin significantly improves its properties, yield, and application [4]. Utilization of natural resources has greater potential because of their abundant availability, low cost, nontoxicity, and biocompatibility. Pectin derived from natural sources has good biodegradability and biocompatible properties; hence, it has great potential in the pharmaceutical and food industries [5].

Extraction is the most important step in pectin production. The most common method of pectin extraction is to use a diluted hot strong mineral acid solvent. However, the drawback is that strong acids are corrosive, impacting health. Strong acid liquid waste must also be processed once more to prevent environmental harm, which raises the expense. Therefore, another alternative that is often used is the extraction of pectin from the skin of fruit using a weak organic acid such as citric acid [6]. *Theobroma cacao* L. (Sterculiaceae) is an important plant in several tropical countries that produces various cocoa-based products. After processing cocoa beans, the cocoa pod husk waste is usually disposed of, causing environmental problems, such as bad odors and disease sources. Increased production of dry cocoa beans in the world has resulted in increased waste of cocoa pod husks [7].

Cocoa pod husks are the main waste from cocoa plantations, producing about 70–75% of the weight of whole fruit. Cocoa pod husks are rich in minerals (especially potassium), fiber (including lignin, cellulose, hemicellulose, and pectin) and antioxidants (e.g., phenolic acids). This waste is usually used as animal feed, as well as a starting material for soap and activated carbon, or returned to the soil for cocoa productivity [8]. The lignocellulosic composition of cocoa bean waste has attracted the attention of the scientific and productive sectors to develop it into higher value-added molecular products. The production of these biomolecules can be applied in the fields of food and feed, agriculture, bioenergy, and other segments [9]. The economic value of cocoa pod husks can be increased through processing, such as fermentation, hydrolysis, combustion, extraction, and synthesis. However, its conversion into high value-added products, especially for the food industry, is limited, as well as its health benefits [10]. It is a challenge to increase the usability and economic value of the pod husks. One of the alternative steps for processing cocoa pod husks is as a raw material for pectin production. The increasing industrial demand for pectin in various products provides a great opportunity for the extraction of pectin from cocoa pod husks. Many studies have been carried out to extract pectin from cocoa pod husks.

Optimization of the pectin extraction from cocoa pods by treating sugar acid (ascorbic acid) using a surface response methodology was carried out to optimize the extraction conditions. The use of sugar acid produced less pectin than the use of chemical acids, but the extracted pectin contained higher galacturonic acid [11]. The results of this study indicated that the use of acid solvents was more effective in extracting pectin. Citric acid was explored in the development of a novel hydrothermal process to extract pectin from cocoa pods. The best results were obtained at 120 °C, for 10 min, with 2% *w*/*v*. Pectin was successfully recovered from as much as 19.3% of the biomass used in the pretreatment with 52.2% degree of methyl esterification and 51.9 mg/g XOS. The hydrothermal processing of cocoa pod husks offers a great opportunity for the synthesis of biomolecules according to the obtained results [12]. On the basis of this study, the use of citric acid in the hydrothermal pretreatment process was proven to be able to recover pectin and has a great opportunity to extract this component from natural sources.

Microwave-assisted extraction of pectin from cocoa pods using oxalic acid was also studied. The reduction in the liquid ratio did not have a significant effect on the extraction results. Increasing the concentration of oxalic acid (lower pH) and the extraction time increased the pectin produced but decreased the degree of esterification. This method can significantly shorten the extraction time compared to conventional methods [13]. Another study used cellulases to extract pectin enzymatically. Optimal parameters were 6.0% concentration of raw material, 40 L·g^−1^ enzyme, and 18.54 h of extraction, these conditions experimentally yielded 10.20 g of pectin/100 g raw material, 52.06 g of galacturonic acid/100 g pectin, and 5.31 g of galacturonic acid/100 g raw material. Enzymatic extraction took substantially longer than the microwave extraction technique [14]. Both studies showed that the use of microwaves was better than conventional and enzymatic methods, in terms of the shorter extraction time.

The conventional extraction process with long operating hours at high temperature has been identified to cause thermal degradation of pectin molecules. Microwave technology application in pectin extraction has shown high potential to expedite the extraction process and produce higher yield [15]. Karbuz and Tugrul [16] compared microwave- and ultrasonic-assisted extraction methods for the extraction of pectin from kiwi peel. The highest pectin yield in the ultrasonic method was 17.30% at 75 °C for 45 min, while the pectin yield in the microwave method was 17.97% at 360 W for 3 min. The microwave-assisted extraction method provided a better yield than conventional and ultrasonic extraction; hence, it can be used as an effective and fast technique for pectin extraction.

The use of microwave-assisted extraction (MAE) for pectin extraction from cocoa pod husks was chosen as an alternative method to maceration, having the advantages of a shorter extraction time, less solvent use, and higher yield. In this study, we applied experimental design and statistical approaches of respond methodology to optimize the microwave-assisted extraction of pectin from the pod husks.

## 2. Results

### 2.1. Model Equation

The model equation of measured parameters is one of the outputs of the optimization process that can be used to predict the response value. Equations (1)–(7) are the prediction models of yield (Y_1_), temperature (Y_2_), water content (Y_3_), ash content (Y_4_), equivalent weight (Y_5_), methoxyl content (Y_6_), and galacturonic acid content (Y_7_).

Y_1_ = 6.72 − 0.632X_1_ + 0.0530X_2_ − 0.000084X_2_^2^ + 0.000987X_1_X_2_. (1)

Y_2_ = 94.47 + 0.238X_1_ + 0.00628X_2_. (2)

Y_3_ = 49.3 − 2.564X_1_ − 0.0289X_2_ + 0.0473X_1_^2^. (3)

Y_4_ = −20.7 + 0.16X_1_ + 0.1030X_2_ − 0.000139X_2_^2^ + 0.1382X_3_^2^. (4)

Y_5_ = 3496 − 60.6X_1_ − 7.00X_2_ − 42.3X_3_ + 0.00496X_2_^2^. (5)

Y_6_ = 2.136 + 0.0305X_1_ + 0.00755X_2_ + 0.0824X_3_. (6)

Y_7_ = 27.8 − 0.73X_1_ + 0.0469X_2_ − 0.14X_3_. (7)

The prediction model above shows the relationship among extraction time (X_1_), microwave power (X_2_), and solvent concentration (X_3_) in terms of the measured parameters. Extraction time had a negative contribution to yield, moisture content, equivalent weight, and galacturonic acid content, whereby a longer extraction time led to lower parameter values. Conversely, extraction time had a positive contribution to temperature, ash content, and methoxyl content, indicating that the value of these parameters increased with increasing extraction time. Microwave power had a positive contribution to yield, temperature, ash content, methoxyl content, and galacturonic acid content but a negative contribution to moisture content and equivalent weight. Solvent concentration showed a directly proportional relationship to temperature, moisture content, and methoxyl content, but an inversely proportional relationship to yield, ash content, equivalent weight, and galacturonic acid content. In addition to linear effects, there were quadratic effects and interactions, as can be seen in the model equations.

A quadratic effect (X^2^) indicates a quadratic relationship which can be represented in a curve (response surface graph), whereby the optimal points of X are on the curve [17]. The extraction time only had a quadratic effect on the moisture content. Microwave power had a quadratic effect on yield, ash content, and equivalent weight. The solvent concentration only had a quadratic effect on the ash content. An interaction effect only occurred for the yield parameter between extraction time and microwave power, indicating that the effect of the extraction time depended on the microwave power [17].

### 2.2. Comparison of Experimental Value and Predicted Value

**Figure 1:**
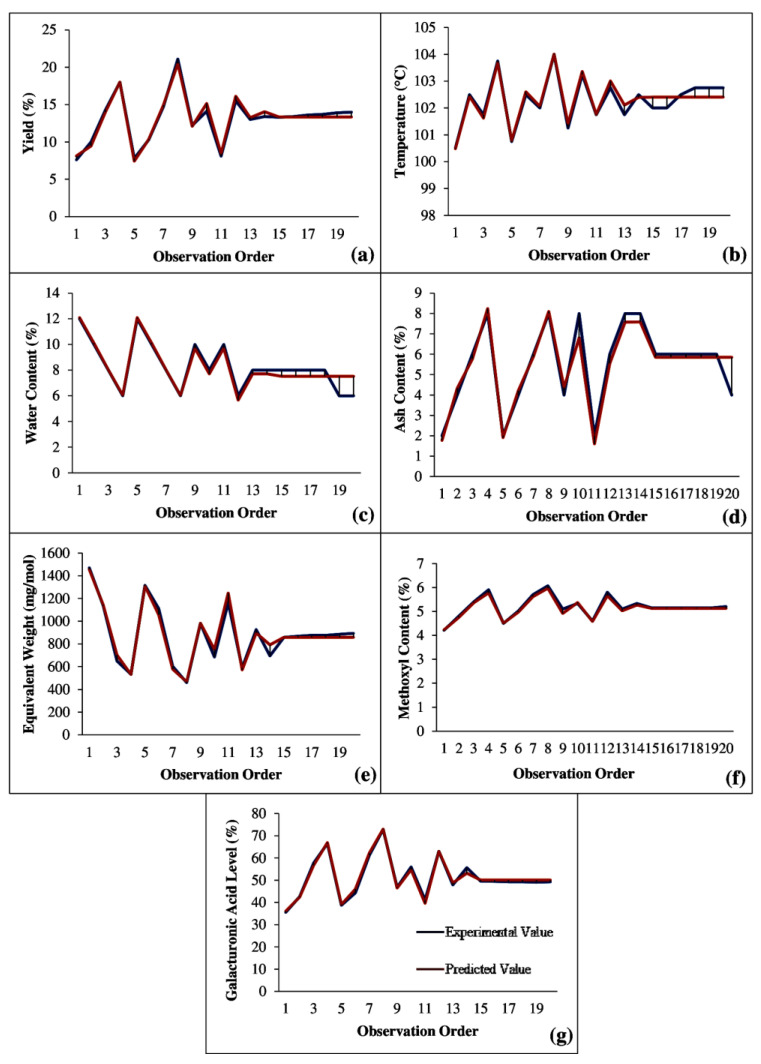
Comparison graph of experimental value and predicted value: (**a**) yield; (**b**) temperature; (**c**) water content; (**d**) ash content; (**e**) equivalent weight; (**f**) methoxyl content; (**g**) galacturonic acid content.

A comparison of experimental values and predicted values obtained from the model equations can be seen in Figure 1. The deviation between the experimental result value and the value from the model equation can be reviewed as a function of model standard deviation and error. From Figure 1, it can be seen that the difference between the experimental value and predicted value was small, which applied to all parameters measured. Model equations for all parameters had a very small error value, as can be seen in Table 1. The error was calculated on the basis of the difference between the square of the observed value and the predicted value divided by the standard deviation. This showed that the equation model generated using the statistical method was acceptable. The interaction among extraction time, microwave power, and solvent concentration was found to significantly affect pectin extraction.

### 2.3. Analysis of Variance (ANOVA)

ANOVA was used to check the significance of the model equation according to two hypotheses [17]:

- H_0_ indicates no interaction effect between independent variables that affects response (parameter).
- H_1_ indicates at least one input variable that affects response.

The hypothesis is established according to the F-value from the ANOVA results. The F-test is performed by comparing the calculated F value with the F_table_ (F_table_ with degrees of freedom 1 (N_1_) = k − 1 = 3 and degrees of freedom 2 (N_2_) = n − k = 16 is 3.24) [18]. The ANOVA results for all parameters are presented in Table 2.

The value of F_count_ for yield was 10.18 (which is greater than F_table_ = 3.24); thus, it can be concluded that there was a significant relationship (regression model was accepted). In terms of the above hypotheses, H_0_ was rejected and H_1_ was accepted, highlighting the influence of the three variables on yield. The F_count_ of temperature was 0.42 (which is smaller than F_table_ = 3.24); thus, it can be concluded that there was no significant relationship (regression model was rejected). In terms of the above hypothesis, H_0_ was accepted and H_1_ was rejected, highlighting that there was no influence of the three variables on temperature. When comparing the calculated F-values of the other parameters, the results showed that there was no influence of the three variables on moisture content and ash content (H_0_ was accepted), but an influence on equivalent weight, methoxyl content, and galacturonic acid content (H_1_ was accepted). The *R*^2^ or coefficient of determination shows the equation model’s accuracy and demonstrates the strength of the independent variables’ influence on the measured parameters.

### 2.4. Lack-of-Fit Test

The suitability of the model equation for each parameter must be tested to see if the model obtained can be used to predict the response value. The assessment in this test is based on the *p*-value for the lack-of-fit model according to two hypotheses:

- H_0_ states that there is no lack of fit (appropriate model).
- H_1_ states that there is a lack of fit (the model does not fit).

The model is considered suitable if the *p*-value is greater than α [17]. Judging from the lack-of-fit *p*-value of all parameters in Table 3, only the models of temperature, moisture content, and ash content were appropriate, where H_0_ was accepted and H_1_ was rejected, in contrast to the model mismatch for other parameters such as yield, equivalent weight, methoxyl content, and galacturonic acid content. Therefore, for further statistical tests, the results of other parameters that were not appropriate were not considered.

### 2.5. Regression Coefficient Test

#### 2.5.1. Individual Regression Coefficient Test

The independent variable is considered to have a significant effect on the parameters measured if the *p*-value is less than the specified significance level value (α) [17]. In this study, the significance level used was 0.05 (α = 0.05), indicating that the error tolerance in this study was 5%. Results of the individual regression coefficient test can be seen in Table 4.

On the basis of the *p*-value of each independent variable on all measured parameters, it was found that extraction time and microwave power had a significant effect on all measured parameters, while solvent concentration did not give a significant effect.

#### 2.5.2. Simultaneous Regression Coefficient Test

According to the results of the simultaneous regression coefficient test, it can be seen in Table 5 that all parameters had a *p*-value less than α (*p*-value < 0.05) for linear regression. These results indicate that the independent variables made a significant contribution to the linear model. For the quadratic model, the independent variables only contributed significantly to the parameters of yield, moisture content, ash content, and equivalent weight, as indicated by the *p*-value less than α. Meanwhile, for other parameters such as temperature, methoxyl content, and galacturonic acid content, independent variables did not have a significant effect on the quadratic model.

### 2.6. Residual Assumption Test

#### 2.6.1. Identical Assumption

**Figure 2:**
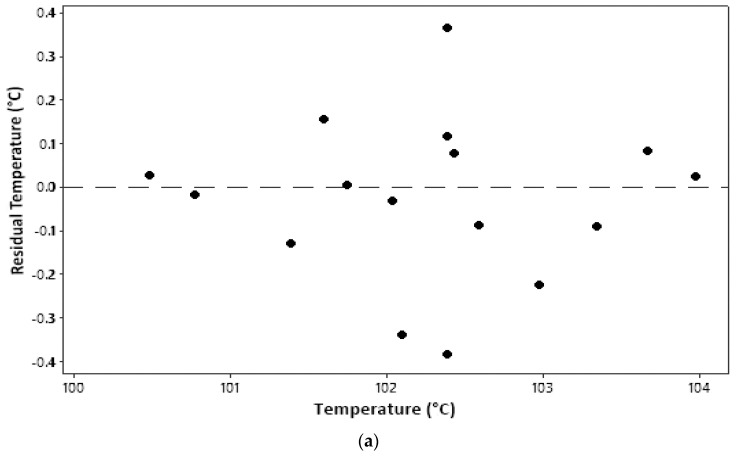

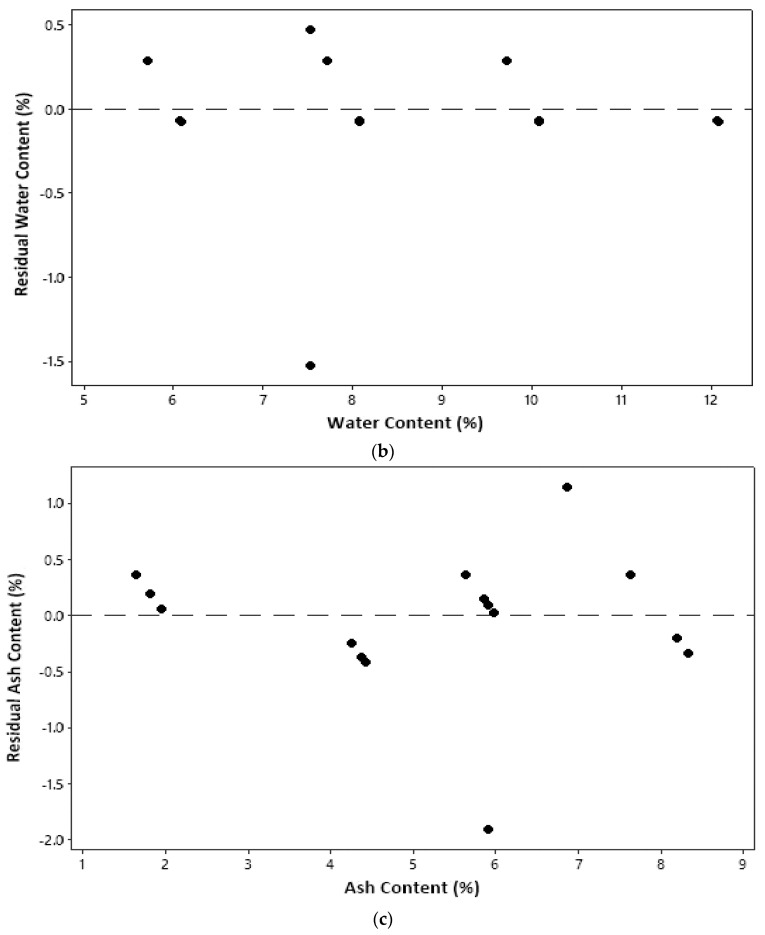
Identical assumption test: (**a**) temperature; (**b**) water content; (**c**) ash content.

An identical assumption test can be seen from a plot between the parameter value and residual, as shown in Figure 2. The residual is an error in measurement obtained from the difference between the experimental value and the model’s predicted value. All plots in Figure 2 show that all model parameters measured qualified for identical assumptions. An even distribution (homoscedasticity) is indicated by residual points that are randomly distributed and do not follow a certain pattern [17].

#### 2.6.2. Independent Assumption

**Figure 3:**
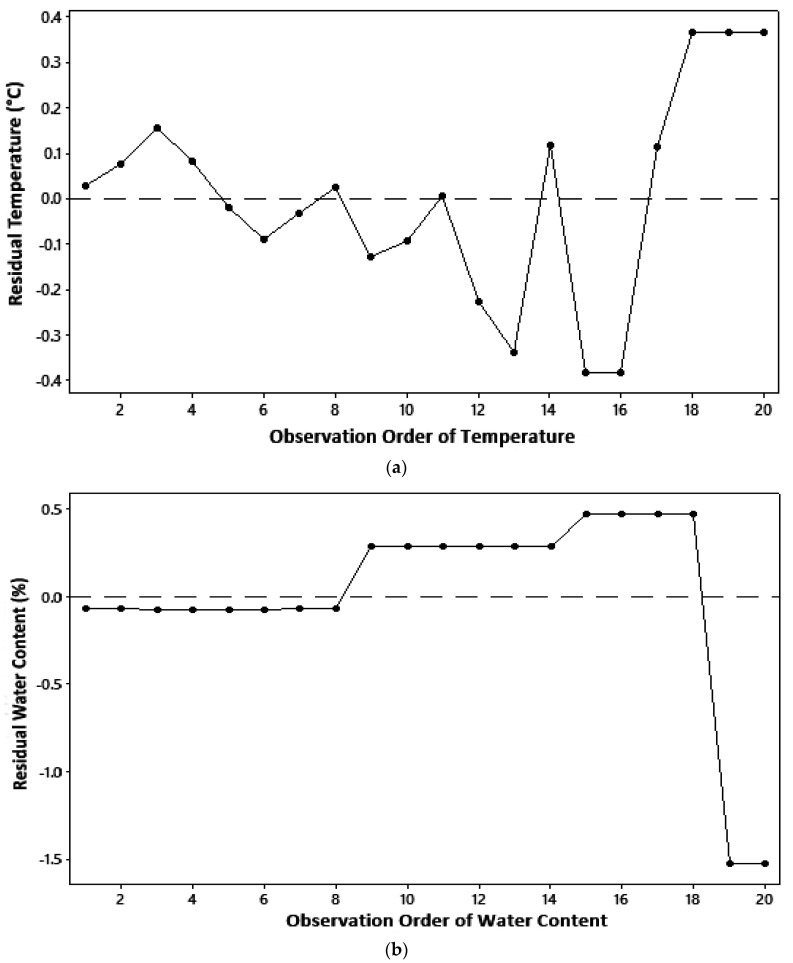

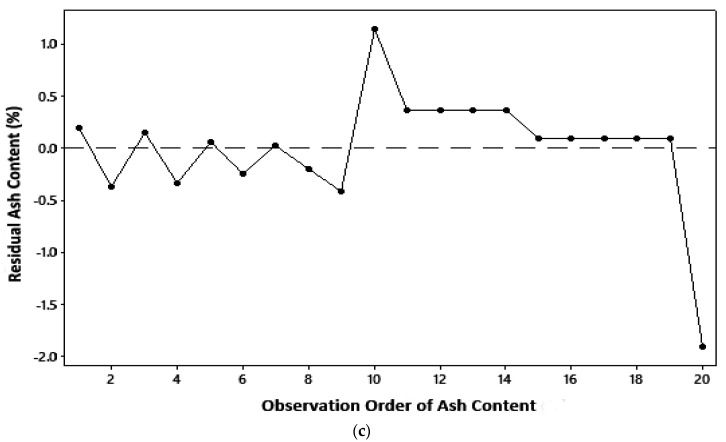
Independent assumption test: (**a**) temperature; (**b**) water content; (**c**) ash content.

The residual of a model must be independent, which states that independent variables are uncorrelated or observations do not depend on a certain time sequence [17]. The independent assumption can be derived from a plot between a sequence of observations and a residual value of observed parameters; as in the identical assumption test, the independence assumption is fulfilled if residual points are randomly distributed and do not form a systematic pattern. On the basis of the distribution of residual points in Figure 3, all parameters measured in this study qualified for independent assumptions.

#### 2.6.3. Normal Distribution Assumption

**Figure 4:**
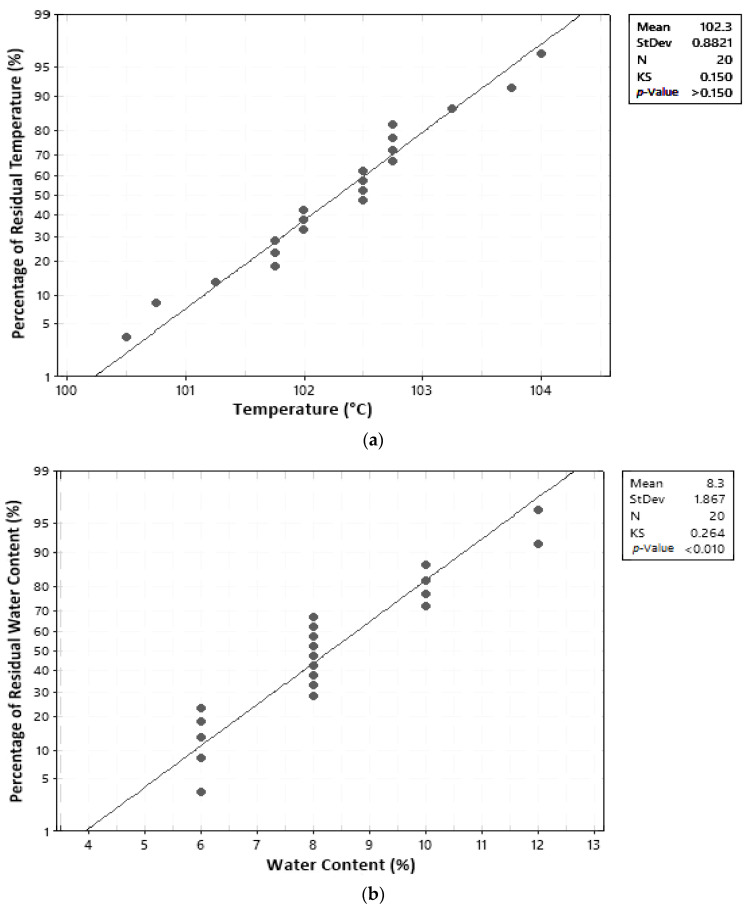

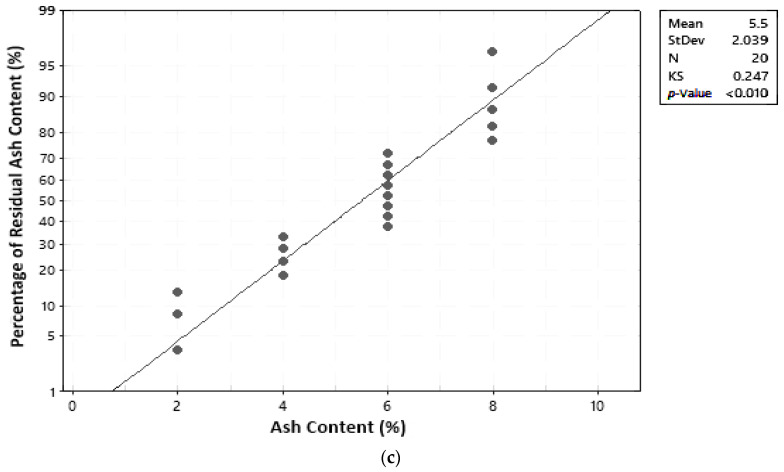
Normal distribution assumption test: (**a**) temperature; (**b**) water content; (**c**) ash content.

In this study, the normal distribution assumption was assessed using graphs and the Kolmogorov–Smirnov (KS) statistical test. A residual is considered to be normally distributed if residual points are close along a straight line and the calculated KS value is less than the table KS value [17]. The KS_table_ for a 5% significance degree and 20 trials is 0.294 [19]. Results of the normal distribution test can be seen in Table 6 and Figure 4. Results were obtained from the normal distribution test based on the graph and Kolmogorov–Smirnov values for temperature, water content, and ash content.

### 2.7. Optimum Conditions of Pectin Extraction from Cocoa Pod Husk Using Microwave Irradiation

Among the seven measured parameters, only three parameter models were suitable for use to predict the response value, as discussed below.

#### 2.7.1. Temperature

**Figure 5:**
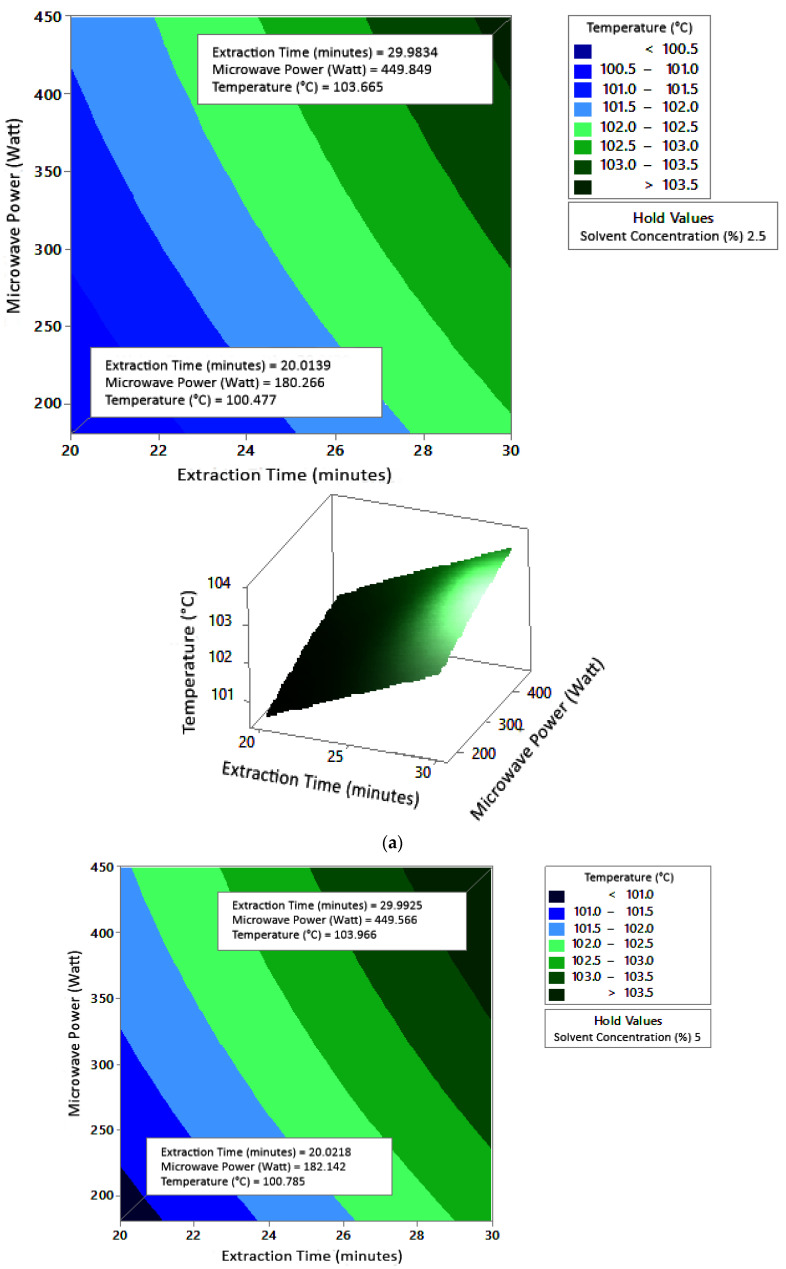

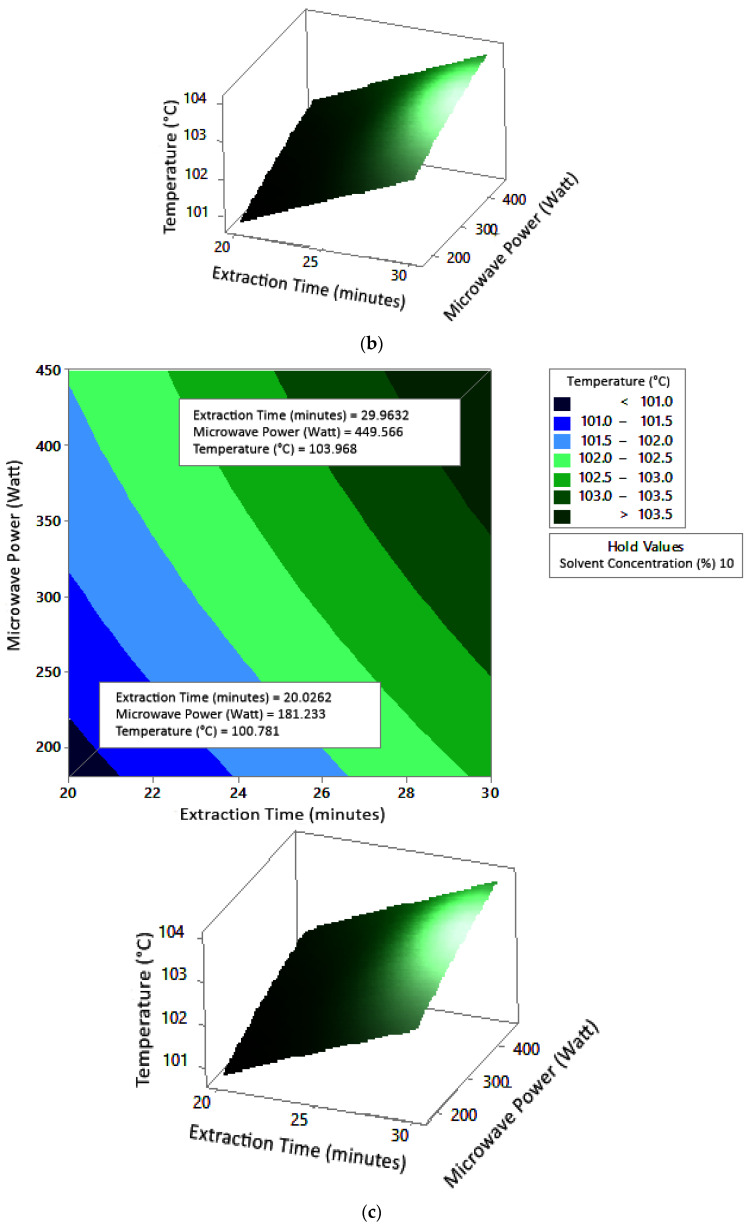
Effect of extraction time and microwave power on temperature at solvent concentrations: (**a**) 2.5%, (**b**) 5%, and (**c**) 10%.

**Figure 6:**
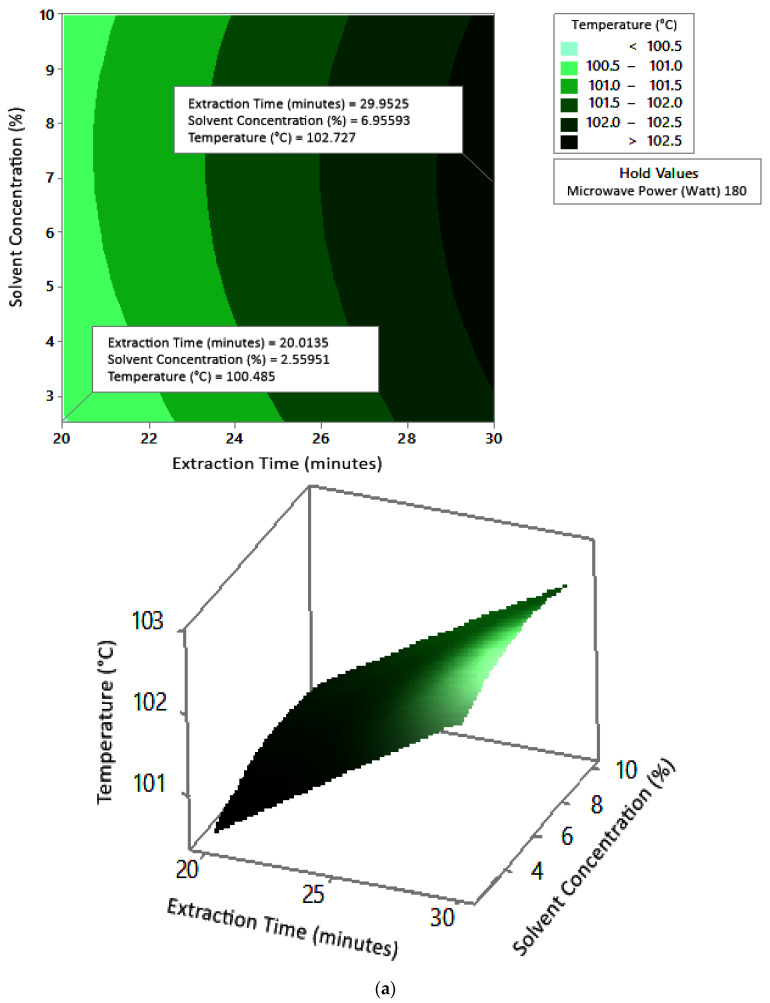

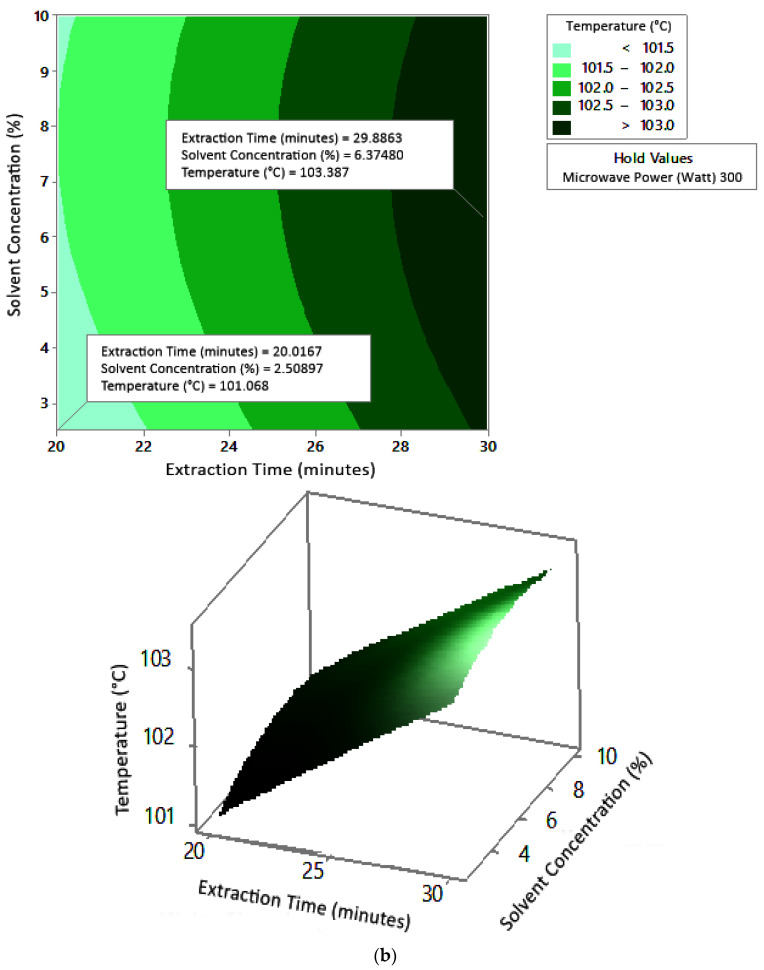

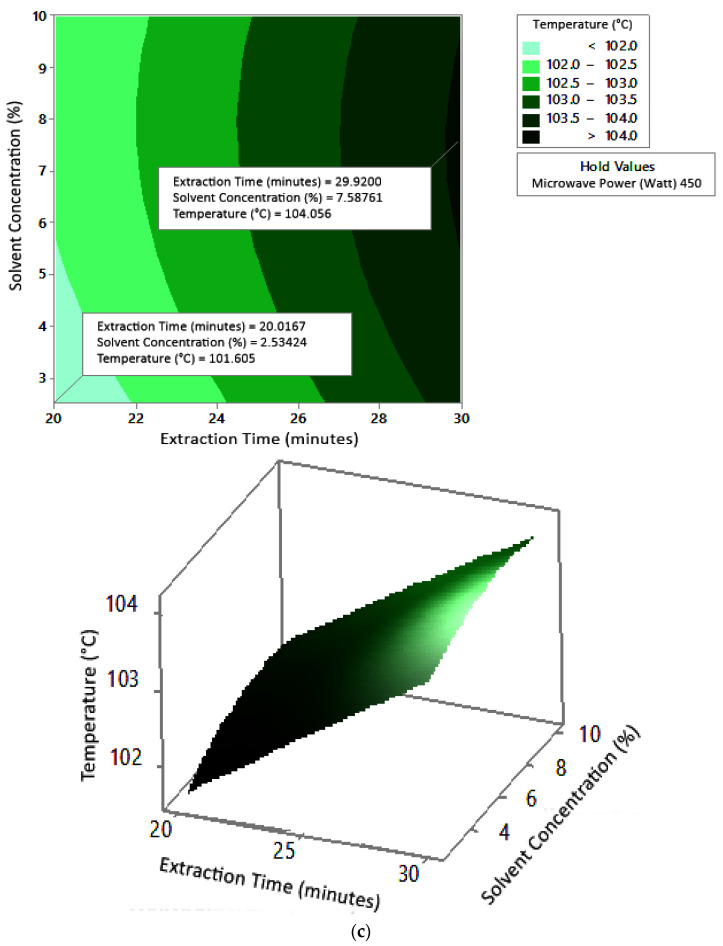
Effect of extraction time and solvent concentration on temperature at microwave powers: (**a**) 180 W, (**b**) 300 W, and (**c**) 450 W.

**Figure 7:**
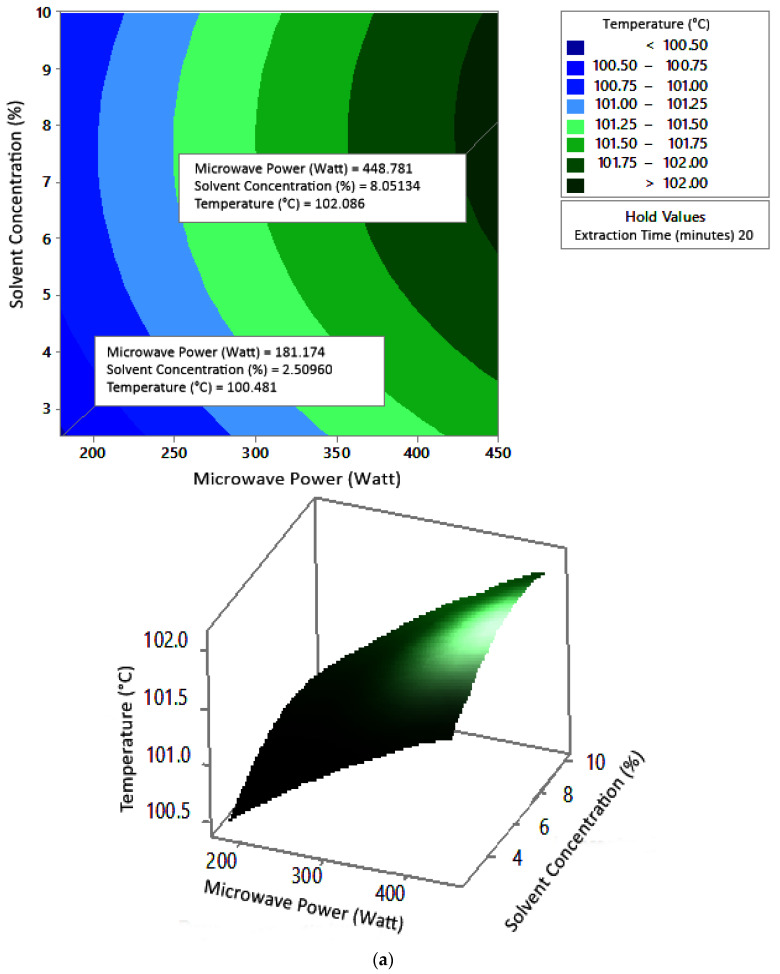

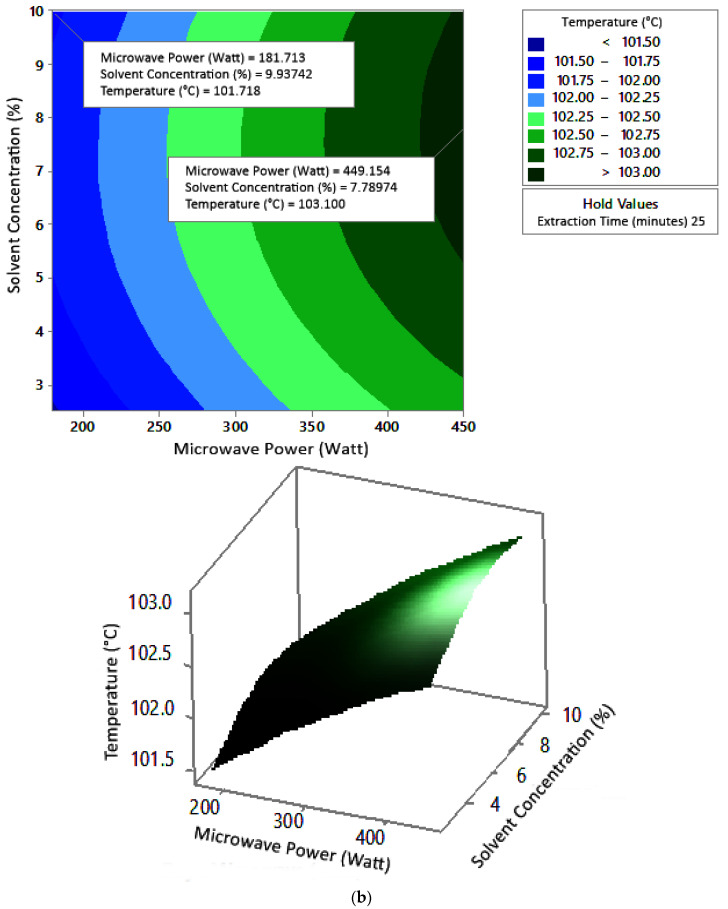

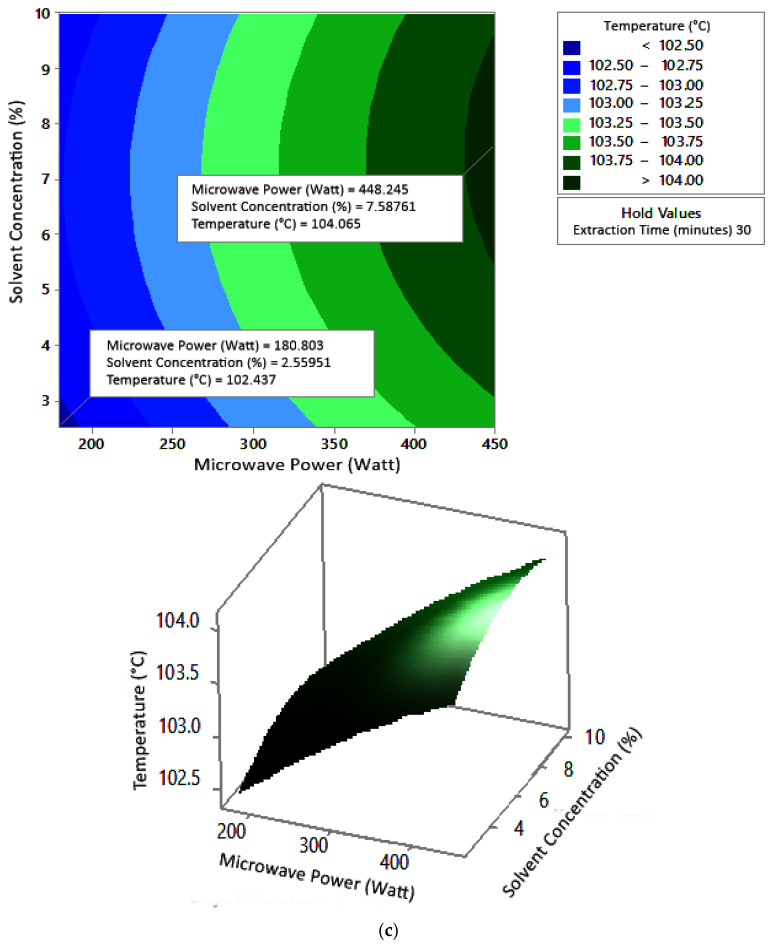
Effect of microwave power and solvent concentration on temperature at extraction times of (**a**) 20 min, (**b**) 25 min, and (**c**) 30 min.

Figure 5 shows the graphs of the effect of extraction time and microwave power on temperature. The temperature increased with increasing extraction time and microwave power, but the temperature range was not too large (approximately ±100–104 °C at each solvent concentration used). Figure 6 shows the graphs of the effect of extraction time and solvent concentration on temperature. With each microwave power used, the temperature increased with the greater solvent concentration and the longer extraction time. Furthermore, the extraction time had a significant effect on increasing the temperature. Figure 7 shows the graphs of the effect of microwave power and solvent concentration on temperature. At each extraction time, the increase in microwave power and solvent concentration was directly proportional to the increase in temperature. As with the previous graph, in this case, the increase in temperature was also significantly affected by the power factor. According to the *p*-value of each variable, solvent concentration did not have a significant effect on temperature.

#### 2.7.2. Water Content

**Figure 8:**
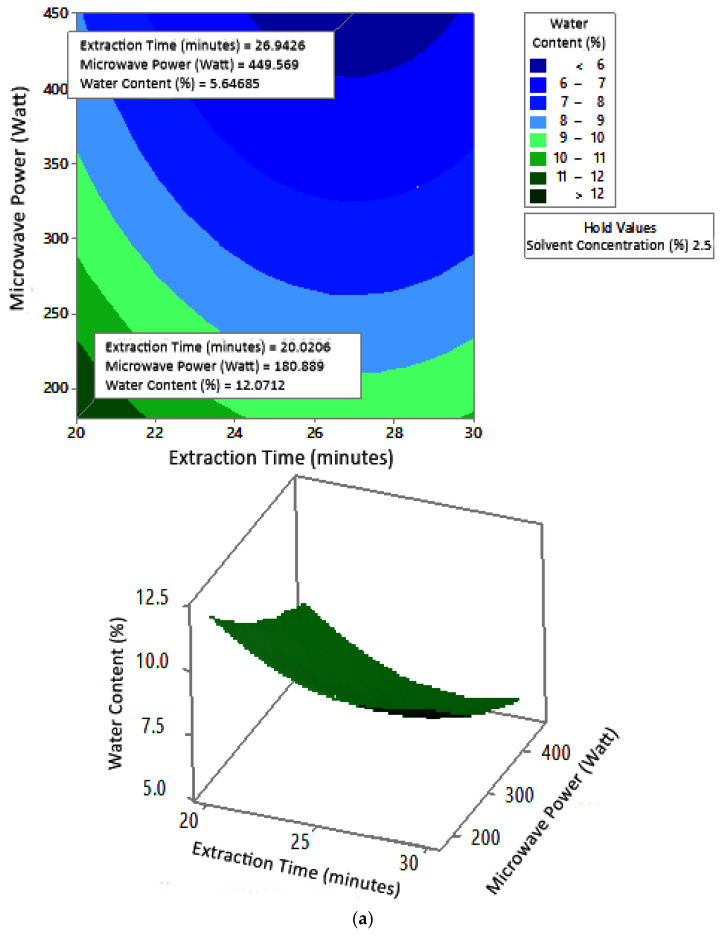

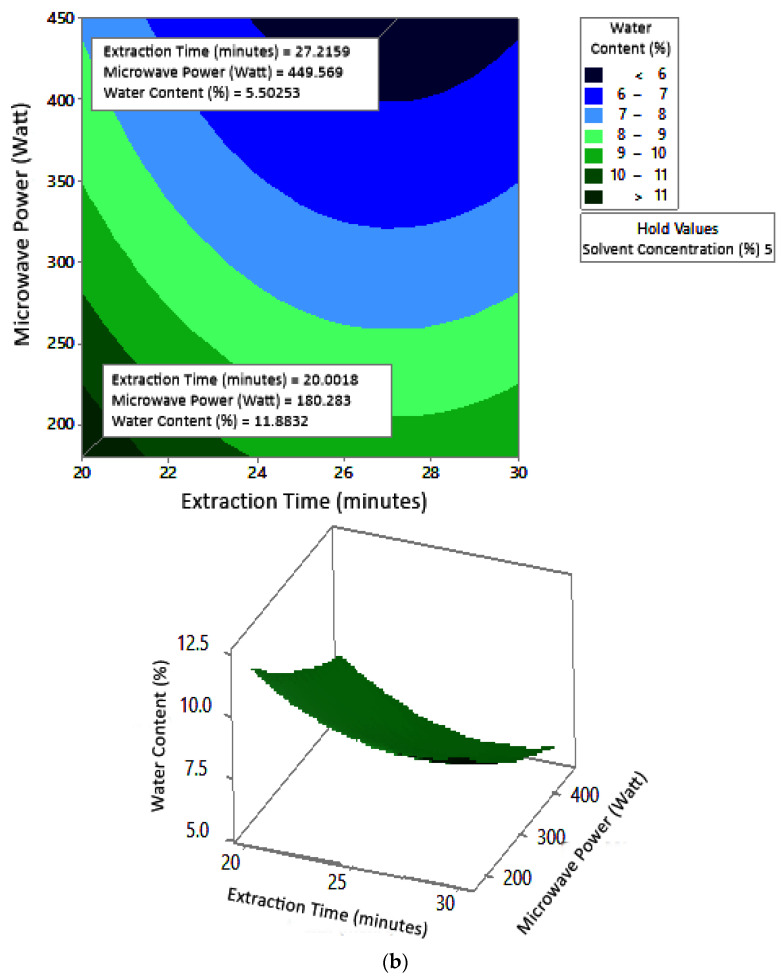

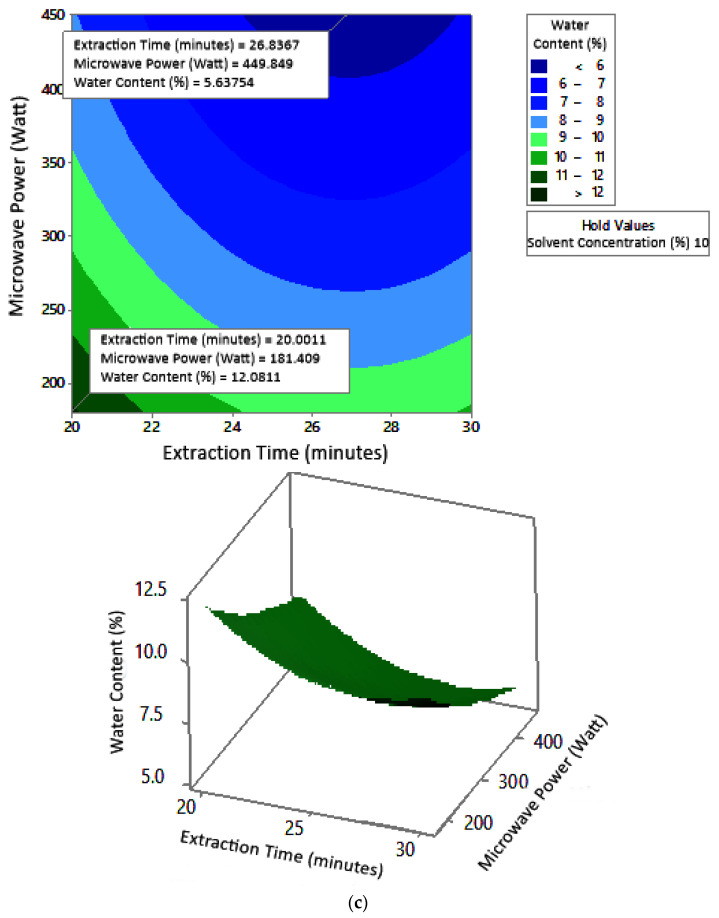
Effect of extraction time and microwave power on water content at solvent concentrations: (**a**) 2.5%, (**b**) 5%, and (**c**) 10%.

**Figure 9:**
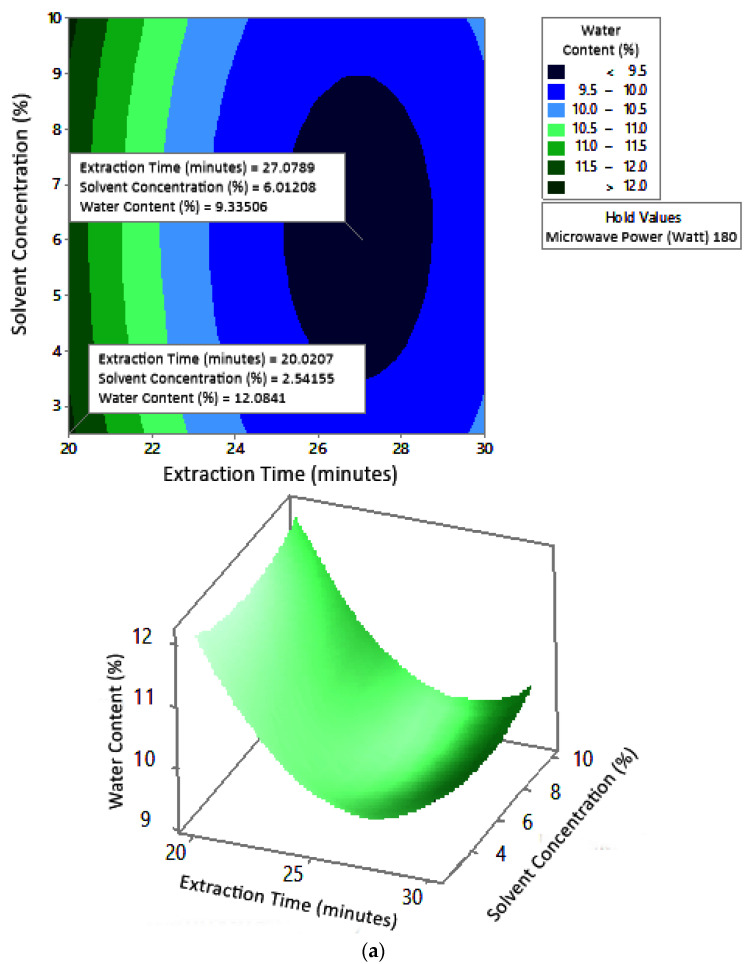

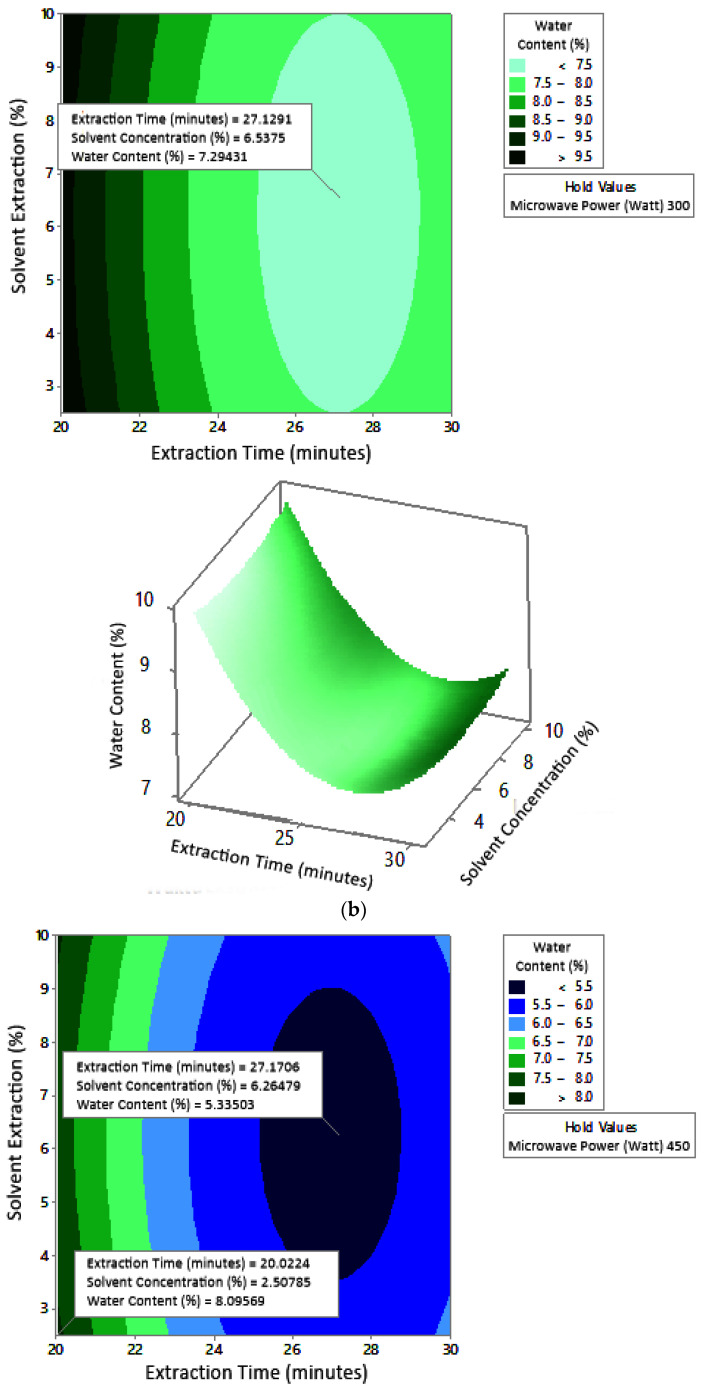

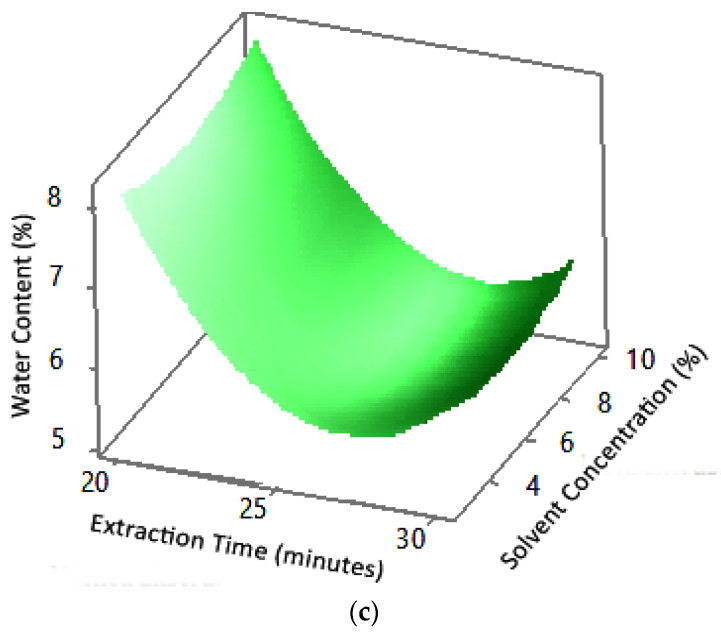
Effect of extraction time and solvent concentration on water content at microwave powers: (**a**) 180 W, (**b**) 300 W, and (**c**) 450 W.

**Figure 10:**
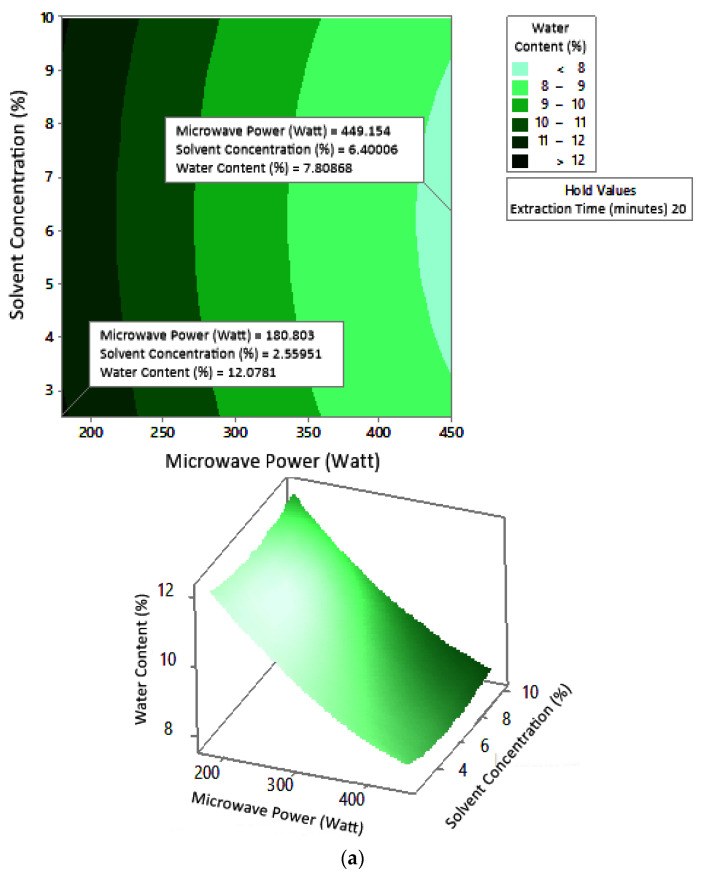

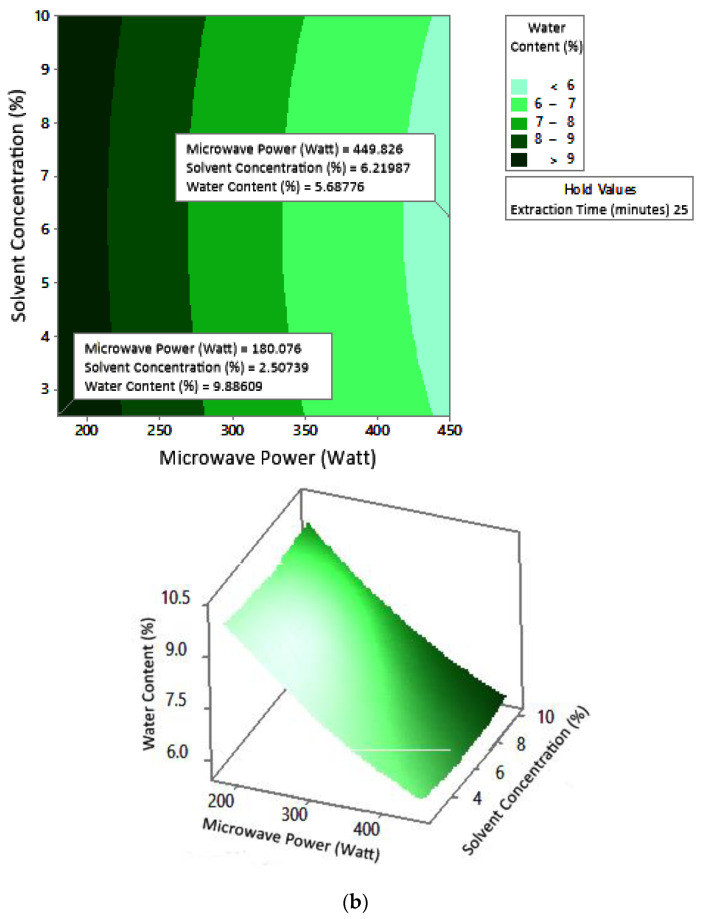

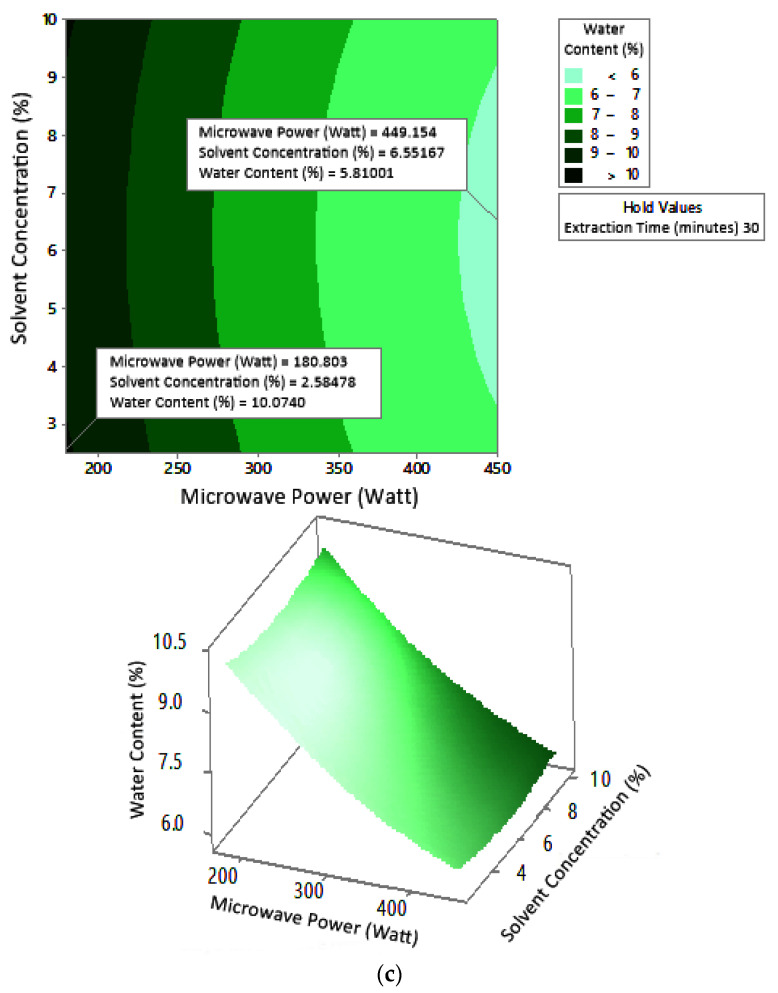
Effect of microwave power and solvent concentration on water content at extraction times: (**a**) 20 min, (**b**) 25 min, and (**c**) 30 min.

Figure 8 shows the graphs of the effect of extraction time and microwave power on water content. The water content decreased with increasing extraction time and microwave power at each solvent concentration used. Figure 9 shows the graphs of the effect of extraction time and solvent concentration on water content. For each microwave power used, water content decreased with the greater solvent concentration and the longer extraction time. Figure 10 shows the graphs of the effect of microwave power and solvent concentration on water content. At each extraction time, the increase in microwave power and solvent concentration was directly proportional to the decrease in water content. Among the three variables, only solvent concentration did not have a significant effect on water content. Thus, it can be assumed that the increase in extraction time and microwave power affected the decrease in water content. 

#### 2.7.3. Ash Content

**Figure 11:**
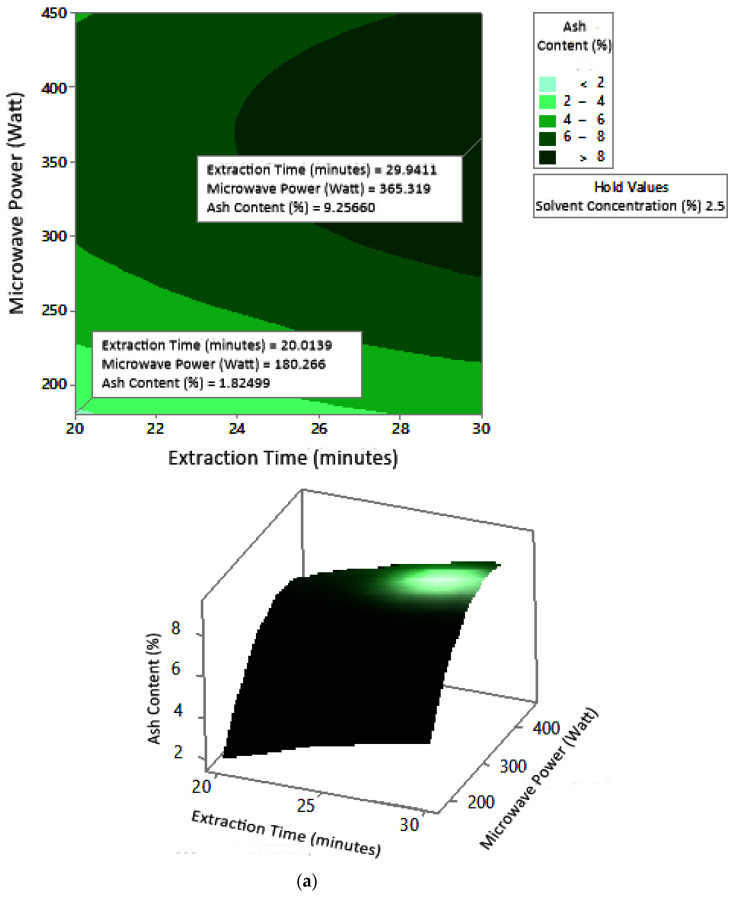

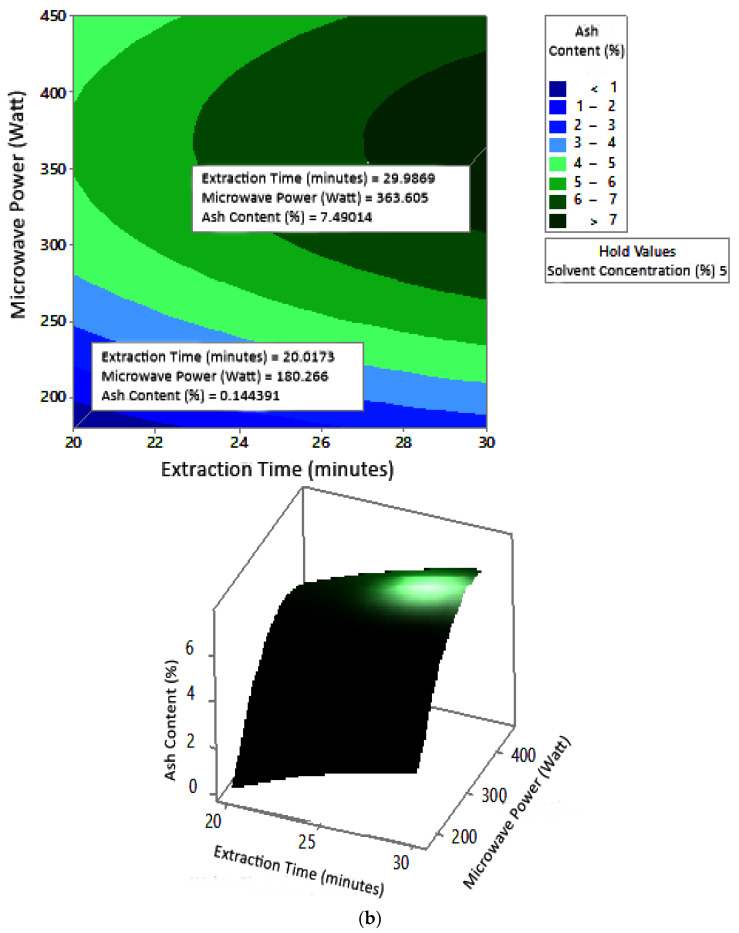

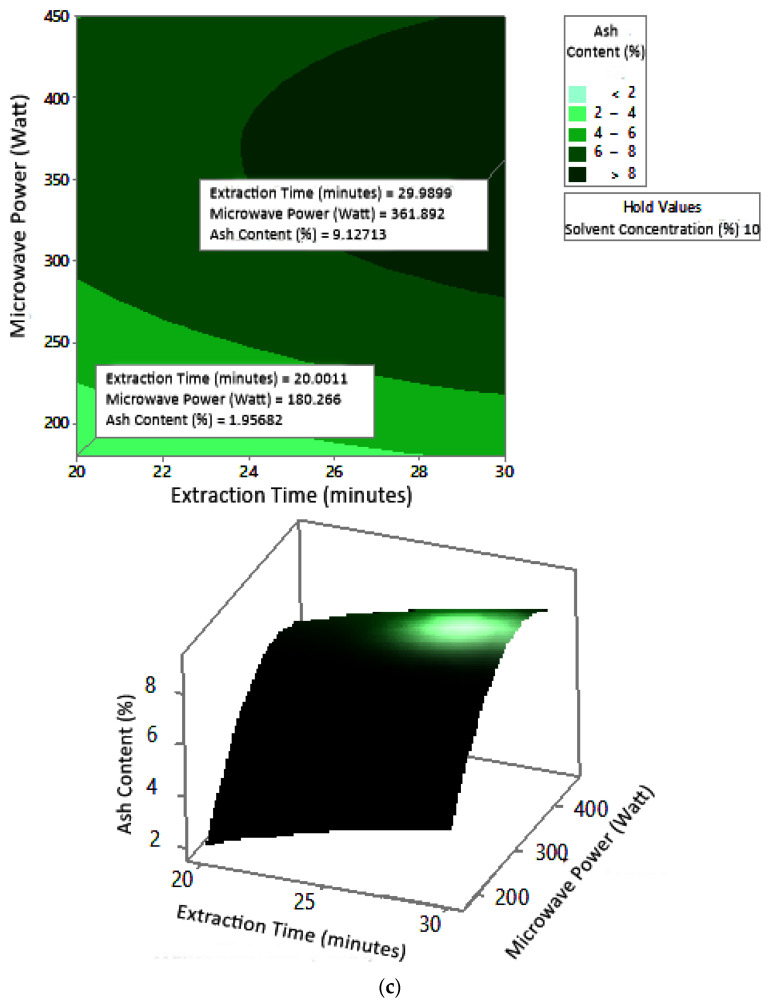
Effect of extraction time and microwave power on ash content at solvent concentrations: (**a**) 2.5%, (**b**) 5%, and (**c**) 10%.

**Figure 12:**
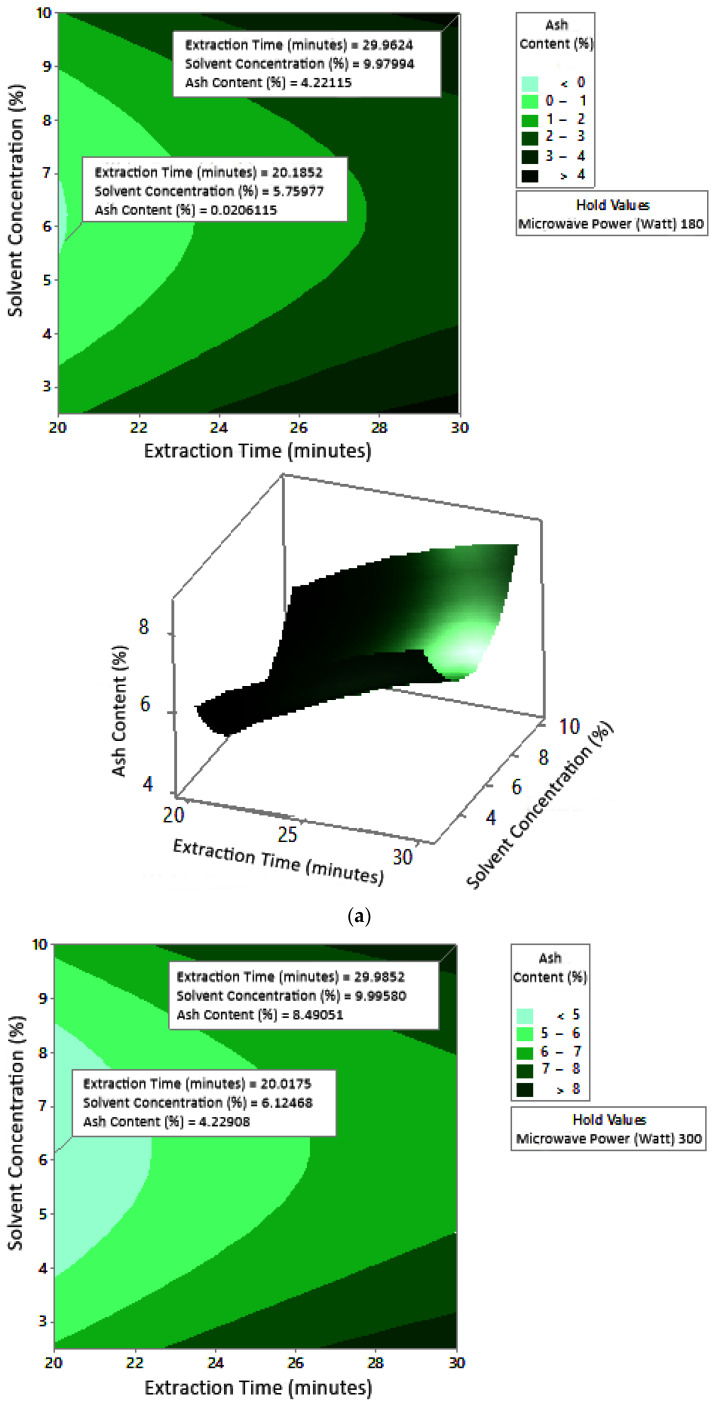

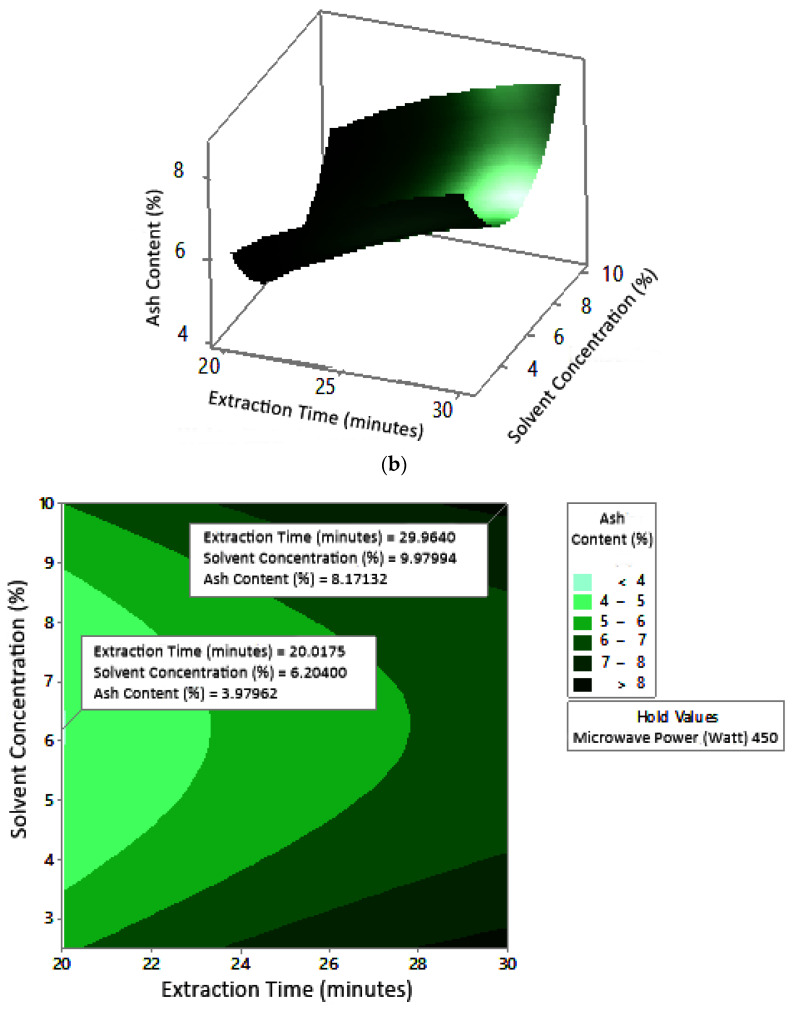

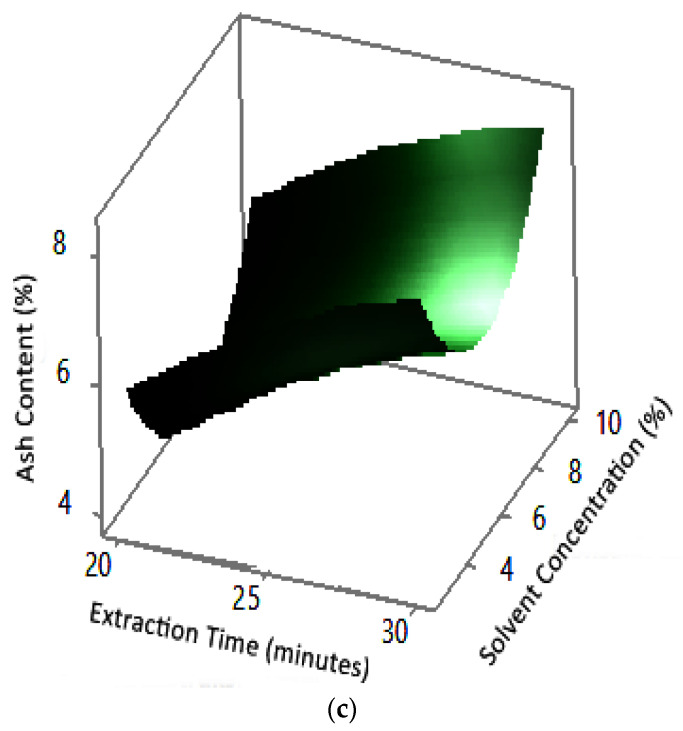
Effect of extraction time and solvent concentration on ash content at microwave powers: (**a**) 180 W, (**b**) 300 W, and (**c**) 450 W.

**Figure 13:**
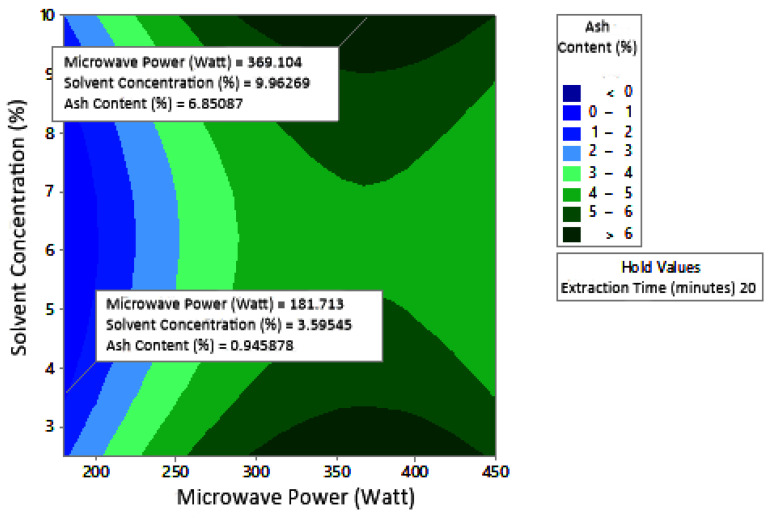

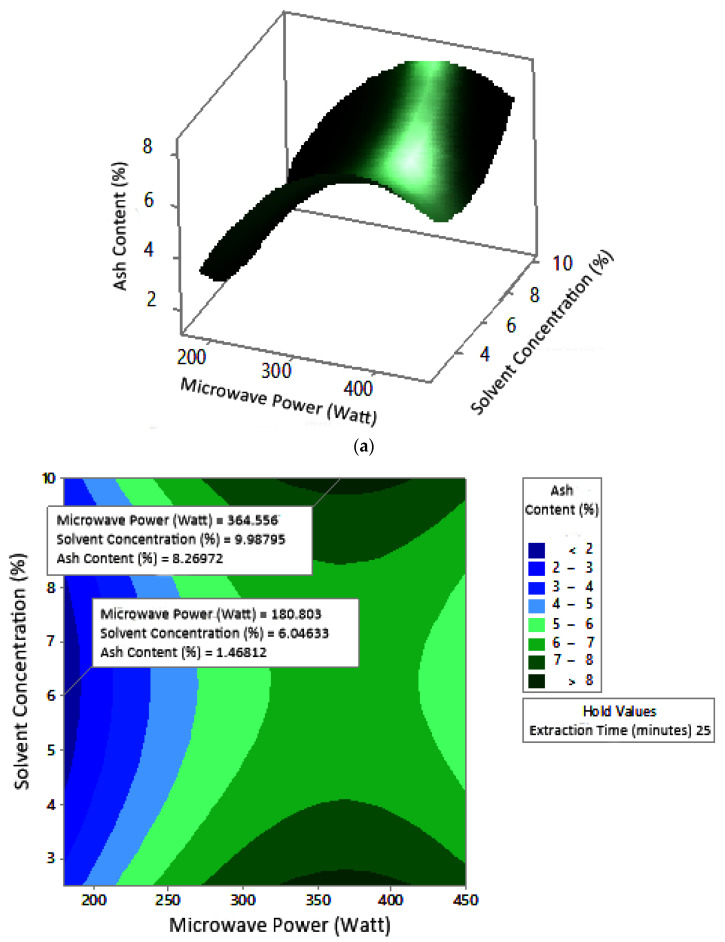

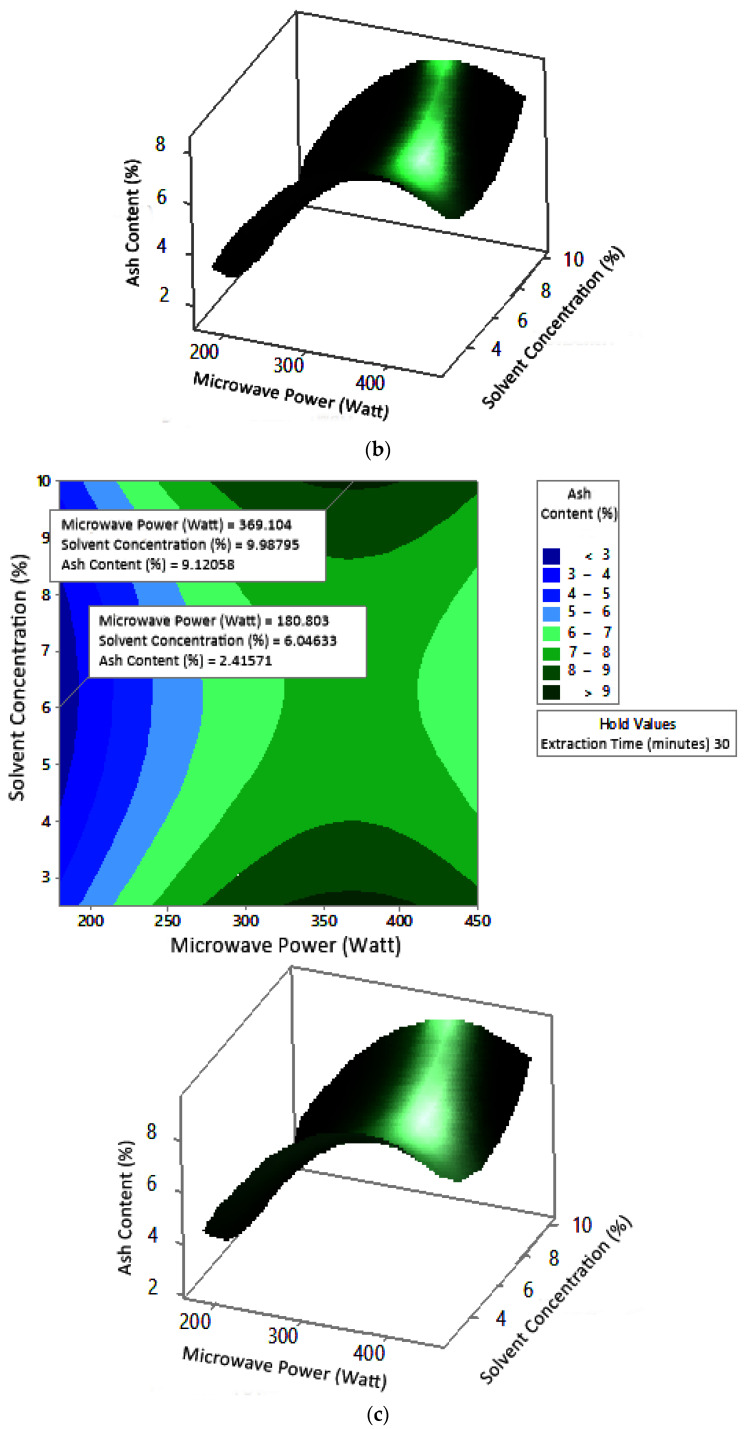
Effect of microwave power and solvent concentration on ash content at extraction times: (**a**) 20 min, (**b**) 25 min, and (**c**) 350 min.

The profile of the effect of extraction time and microwave power on ash content at certain solvent concentrations can be seen in Figure 11. Ash content fluctuated with increasing microwave power and a longer extraction time. The effect of extraction time and solvent concentration on specific microwave power can be seen in Figure 12. Ash content was directly proportional to the increase in extraction time and solvent concentration. Figure 13 shows the effect of microwave power and solvent concentration at a fixed extraction time. Ash content was also directly proportional to the increase in microwave power and solvent concentration. However, as with the previous parameter, the solvent concentration had no significant effect on the ash content. Overall, only extraction time and microwave power affected the ash content.

## 3. Discussion

### 3.1. Temperature

The optimum conditions for temperature were determined on the basis of Figure 5 because the variable solvent concentration had the smallest effect among all independent variables (*p*-value = 0.155). Figure 5 shows three-dimensional plots of temperature as a function of time and power at 2.5%, 5%, and 10% concentrations of citric acid. At a constant concentration, the temperature increased with a longer extraction time and greater microwave power. This occurred in all variations of solvent concentration. Figure 5a–c show that the maximum temperature was around 104 °C, at an extraction time of 30 min and a microwave power of 450 W. The minimum temperature was around 100 °C, obtained at an extraction time of 20 min and a microwave power of approximately 180–182 W.

Although the temperature was a measured parameter, in this discussion, we relate it to the pectin yield obtained because temperature also affected the yield in the extraction process. The effect given by the extraction time and microwave power on temperature also indirectly affected the pectin yield. A longer extraction time and a greater microwave power led to an increase in temperature, which caused an increase in the pectin yield. Phaiphan et al. [20] found that pectin yield and chemical characteristics increased with increasing extraction time and microwave power. The pectin yield obtained was 20.93% to 22.91% at 300 W microwave power for 5–15 min.

A comparative study of the pectin extraction from watermelon peel with conventional and microwave-assisted heating conducted by Jiang et al. [21] showed that the yield of pectin in microwave-assisted extraction was higher than the yield in conventional extraction. The average yield in conventional extraction was 17.5% under the optimum conditions, which were a temperature of 90 °C, pH of 1.0, time of 150 min, and solid–liquid ratio of 1:20. Meanwhile, the average yield for microwave-assisted extraction was 19.3%, obtained at the best parameter conditions of a power of 500 W, pH of 1.5, time of 7 min, and solid–liquid ratio of 1:10.

In order to extract pectin from plants, increased microwave power can improve solvent penetration into the plant matrix. Polar solvents such as water are effective in absorbing microwave energy and producing heat. Microwave irradiation accelerates the rupture of plant cells via a sudden increase in temperature and an increase in internal pressure within the plant cell, which promotes the destruction of the surface of the sample. This helps the process of releasing pectin in plant cells to the solvent and its surroundings, which increases the extraction yield. However, prolonged exposure to microwaves can cause pectin degradation [22].

The yield of pectin from cocoa pod husk extraction using conventional heating with ascorbic acid solvent produced a yield of 4.2% ± 0.12% at a temperature of 95 °C, pH of 2.5, and time of 45 min [11]. The extraction of pectin from cocoa pod husks with citric acid on conventional heating resulted in a pectin yield of 10.1% under conditions of a temperature of 95 °C, pH of 3.0, and time of 95 min [23]. Another study that extracted cocoa pod husks with citric acid using the conventional method resulted in a pectin yield of 7.62% at a temperature of 95 °C, pH of 2.5, and time of 3 h [24].

According to the results of studies conducted with as many as 20 experiments, the measured temperature range was 100.5 to 104 °C. The highest temperature (104 °C) was measured for 30 min, 450 W, and 10% citric acid, obtaining a pectin yield of 21.1%. Conditions that produced maximum yield were considered optimum conditions. This value was the maximum experimental pectin yield obtained in this study. Although the process took place at a lower temperature, the yield produced in conventional extraction was quite small despite a long extraction time. The use of microwaves in the extraction of pectin from cocoa pod husks solved this problem, whereby a much greater yield was produced in a much shorter time, even though the temperature was slightly higher.

According to the literature, the pectin content in cacao pod husk ranges from 11% to 15% on a dry basis [25]. Therefore, in this study of pectin extraction from cocoa pod husk, more pectin was successfully obtained than the standard pectin yield. This is in accordance with the predicted condition shown by the response surface graph.

### 3.2. Water Content

The optimum conditions for temperature was determined on the basis of Figure 8 because the variable solvent concentration had the smallest effect among all independent variables (*p*-value = 1.000). Figure 8 shows the three-dimensional plots of water content as a function of extraction time and microwave power at solvent concentrations of 2.5%, 5%, and 10%. The minimum water content was approximately 5.6% with an extraction time of about 27 min and a power of 180 W, for all concentration variations. The maximum water content shown by the plot was about 12%, which was obtained using 20 min of extraction with a microwave power of 180 W for all concentration variations. In the extraction time range of 20–30 min and microwave power range of 180–450 W, the moisture content enhanced with increasing time and power. The solvent concentration had no effect on the pectin water content for the extraction time of 20–30 min and the microwave power of 180–450 W.

The water content of pectin obtained from microwave-assisted extraction in this study ranged from 6% to 12%. The experimental maximum water content was obtained for 20 min, 180 W, and 2.5% citric acid, according to the conditions indicated by the response surface graph. The maximum limit of pectin water content according to the FCC (Food Chemical Codex) (1996) is 12% [26]. At maximum conditions, the experimental water content did not exceed the standard. This indicated that microwave-assisted pectin extraction for 20–30 min, with a microwave power of 180–450 W and solvent concentration of 2.5–10%, was able to produce water content according to the FCC standard.

The maximum water content of pectin from conventional extraction of orange peel was 15.03%. This value was obtained in extraction conditions of 80 °C for 120 min with 1 N hydrochloric acid as a solvent [27]. The water content obtained in the extraction of pectin from cocoa pod husks using 0.1 N hydrochloric acid solvent with conventional heating ranged from 10.56% to 11.96% at a temperature of 65–95 °C, pH of 1.5–3, and time of 40–80 min [28]. Although the water content of pectin in conventional extraction also produced water content according to the standard, the extraction time required was longer. The use of microwaves can shorten the extraction time and produce standard water content.

Water content affects the shelf life of pectin by increasing the oxidation and hydrolysis of pectin due to microbial activity [29]. The water content of pectin needs to be kept to a minimum so that its storage is safe and inhibits the growth of microorganisms. Water content affects the growth of microorganisms that can produce pectinase enzymes, thereby affecting the quality of pectin [30].

According to these results, the use of microwaves was more advantageous in pectin extraction because it produced pectin with a maximum water content that was still in accordance with the standard in less intense conditions.

### 3.3. Ash Content

Ash content indicates the total mineral content in the biomass and is used to determine the chemical composition of the sample. Pectin ash content is expected to be low in order to have good gelling ability [15]. The optimum condition for ash content is shown in Figure 11, where solvent concentration was used as the benchmark (*p*-value = 1.000) because it had the least contribution to ash content compared to other variables. Figure 11 presents the three-dimensional plot of ash content as a function of extraction time and microwave power. Figure 11 shows that ash content was directly proportional to the extraction time and microwave power in a time range of 20–30 min and a power range of 180–450 W. The minimum ash content was approximately 0.14% under the extraction conditions of a time of 20 min, power of 180 W, and solvent concentration of 5%. The maximum ash content was 9.26%, which was obtained via extraction for 30 min, with a power of 365 W and solvent concentration of 2.5%.

The experimental ash content in this study ranged from 2% to 8%. The maximum value was obtained in various conditions, namely, 30 min, with a power of 300 W and 450 W (at all citric acid concentrations), and 25 min, with a power 300 W and citric acid concentrations of 2.5% and 10%. The experimental minimum ash content was 2%, obtained at three different experimental conditions, namely, 20 min, 180 W, 2.5% solvent, 20 min, 180 W, 5% solvent, and 25 min, 180 W, 5% solvent.

Ash content in pectin should preferably be less than 10% [31]. The pectin ash content obtained by Souhoka et al. [32] in the extraction of pectin from cocoa pods was 10.634%. These results were obtained under extraction conditions of 5% HCl at 95 °C for 2 h.

The ash content of pectin from seed watermelon peel extraction using conventional and microwave-assisted methods was almost the same (1.7% and 1.6%, respectively). Conditions in the conventional method were a temperature of 90 °C, pH of 1.0, time of 150 min, and solid–liquid ratio of 1:20. Microwave-assisted extraction conditions were carried out at a power of 500 W, pH of 1.5, time of 7 min, and solid–liquid ratio of 1:20 [21]. The extraction of pectin from cocoa pods with 0.1 N hydrochloric acid solvent using the conventional heating method resulted in an ash content of 6.82–8.97%. Extraction was carried out at a temperature of 65–95 °C, pH of 1.5–3, and time of 40–80 min [28]. The ash content in pectin increased with increasing microwave power at constant extraction time [29]. Ash content is important because it affects the ability of pectin to form a gel [30].

According to the results of this study, microwave-assisted pectin extraction produced a lower ash content in a much shorter operating time than the conventional method. Ash content indicates the purity of pectin, whereby a lower pectin ash content denotes higher pectin purity. Thus, the ash content obtained from all experiments met the standard.

Among all measured parameters, solvent concentration did not have a significant effect on the microwave-assisted extraction of pectin from cocoa pod husks. It is possible that the concentration of the solvent does not have an individual effect but an interactive effect with other independent variables not used in this study. A previous study examined the effect of pH, temperature, and time on the extraction of nitric acid pectin from cocoa pods. The results showed that pH and temperature had a more significant effect. It was found that the optimal condition was a pH of 1.5 at 100 °C for 30 min, with pectin yield and uronic acid content predicted at 9.5% and 80%, respectively. This study found that the uronic acid content was higher when the pH was lowered and the temperature was raised, or when the pH was raised and the temperature was lowered [23]. Mellinas et al. [33] observed that pH had a major influence on the microwave-assisted extraction of cocoa pods, whereby alkaline conditions resulted in a higher extraction yield and antioxidant capacity. According to these two studies, pectin extraction was superior under alkaline conditions or at a low pH.

## 4. Materials and Methods

### 4.1. Materials

The main ingredients used for pectin extraction from cocoa pod husk were cocoa pod husk (*Theobroma cacao* L.) as a source of pectin, citric acid as solvent, 96% ethanol, silver nitrate solution, and aquadest. The cocoa pod husks were obtained from a cocoa plantation in Medan Tembung Pasar IX. The cocoa pod husks used were yellow-orange in color before being extracted, drying to a brown color. Additional materials used in this study were sodium chloride, sodium hydroxide, phenolphthalein, calcium chloride, and acetic acid (glacial) 100%. All chemicals used in this study were supplied by Merck. Extraction was carried out in a modified microwave oven (Samsung ME731K 800 W 20 L, PT Samsung Electronic Indonesia, Bekasi, West Java, Indonesia) equipped with a single neck flask, condenser, and thermocouple, while extract filtering was carried out using filter paper (Whatman no. 1 diameter 110 mm, CV Rudang, Medan, Indonesia).

### 4.2. Experimental Design

According to a prior study by Sarah et al. (2018) [34], it was discovered that 30 min of pectin extraction utilizing 300 W of microwave radiation generated the maximum pectin yield. During the extraction time of 10–30 min, the pectin yield fluctuated, but the pectin yield increased in the 20th to 30th minutes. The use of 180 to 450 W microwave power resulted in an increase in the pectin yield. Therefore, in this study, the optimization of microwave-assisted pectin extraction was carried out using an extraction time of 20–30 min and a power of 180–450 W.

For optimization, the experimental response surface method of the second order was used, with a central composite design (CCD). Experimental designs and data processing were achieved using Minitab 17 trial version software. The total number of experiments was 20 consisting of eight factorial points, six central points, and six axial points. The experimental design is shown in Table 7.

### 4.3. Pectin Extraction

Pretreatment and microwave-assisted pectin extraction methods followed the procedure of Sarah et al. [34] with some modifications. Cocoa pod husk was diced to a thickness of about 0.5 cm to facilitate the drying and grinding process. Furthermore, cocoa pod husk was dried in a drying oven at a temperature of 40 °C until the weight was constant, and the moisture content of cocoa pod husk was calculated with a moisture content of 7.9%. Then dried cocoa skin was mashed and sieved using a 60/70 mesh. A total of 10 g of cocoa pod husk powder was extracted using 150 mL of citric acid solvent, where the ratio of the pods to the solvent was 1:15 (*w*/*v*). Extraction was carried out in a microwave with various extraction times (20, 25, and 30 min), microwave powers (180, 300, and 450 W), and solvent concentrations (2.5%, 5%, and 10%). Temperature measurements were carried out during the extraction using a thermocouple mounted on the top of the microwave oven which penetrated the oven and measured the temperature of the extraction solvent. The extracted mixture was then filtered using filter paper to separate the filtrate from the dregs. Then the filtrate was heated at 95 °C while stirring until the volume was half the original volume.

The concentrated filtrate was cooled and then precipitated by adding acidified ethanol (adding 2 mL hydrochloric acid per 1 L of ethanol). The ratio of the filtrate to the added ethanol was 1:1.5. The deposition process was carried out for 16 h. The pectin precipitate was filtered using filter paper to separate it from the ethanol and water solution. The pectin precipitate was washed with ethanol until chloride as removed. The pectin precipitate was separated from the washing liquid using filter paper. To determine the presence of chloride, a few drops of silver nitrate solution (AgNO_3_) were added to the washing liquid. A white precipitate (AgCl) was formed if chloride was still present. Lastly, the pectin precipitate was dried in an oven at 40 °C for 8 h.

### 4.4. Pectin Characterization

#### 4.4.1. Water Content

Water content was determined by drying the pectin in an oven at 105 °C for 2 h until its weight was constant. Water content was calculated using Equation (8) [35].

(8) $$\mathrm{Water}\;\mathrm{Content}\;\left(\%\right)=\frac{W_{2}\;-\;W_{3}}{W_{2}\;-\;W_{1}}\;\times \;100\%,$$

where W_1_ is the weight of the empty plate, W_2_ is the weight of the plate filled with pectin, and W_3_ is the weight of the plate and pectin dried to a constant weight.

#### 4.4.2. Ash Content

The ash content of pectin was determined by drying the pectin in a furnace at 600 °C for 3 h until its weight was constant. Ash content was calculated using Equation (9) [35].

(9) $$\mathrm{Ash}\;\mathrm{Content}\;\left(\%\right)=\frac{W_{3}\;-\;W_{1}}{W_{2}\;-\;W_{1}}\;\times \;100\%,$$

where W_1_ is the weight of the empty plate, W_2_ is the weight of the plate filled with pectin, and W_3_ is the weight of the plate and pectin dried to a constant weight.

#### 4.4.3. Equivalent Weight

Pectin (0.5 g) was moistened with 5 mL of ethanol and dissolved in 100 mL of distilled water, to which 1 g NaCl was added. The resulting mixture was titrated slowly using 0.1 N NaOH until the phenolphthalein indicator changed to rosy red (pH 7.5) which took 30 s. The data obtained were calculated using Equation (10) [35].

(10) $$\mathrm{Equivalent}\;\mathrm{Weight}\;\left(\mathrm{mg}/\mathrm{mol}\right)=\frac{\mathrm{Sample}\;\mathrm{Weight}\;(\mathrm{mg})}{\mathrm{mL}\;\mathrm{NaOH}\;\times \;N\;\mathrm{NaOH}}\;\times \;100\%.$$

#### 4.4.4. Methoxyl Content and Galacturonic Acid Content

The methoxyl and galacturonic acid content was determined using dried pectin. The determination of methoxyl content was carried out by adding 25 mL of 0.25 N NaOH, stirring, and leaving for 30 min at room temperature in a closed state. Furthermore, 25 mL of 0.25 N HCl solution was added and titrated with 0.1 N NaOH until the phenolphthalein indicator turned red. The galacturonic acid content was calculated from the NaOH volume (mL) obtained from the determination of equivalent weight and methoxyl content. The methoxyl and galacturonic acid content was calculated using Equations (11) and (12) [35].

(11) $$\mathrm{Methoxyl}\;\mathrm{Content}\;\left(\%\right)=\frac{\mathrm{mL}\;\mathrm{NaOH}\;\times \;31\;\times \;N\;\mathrm{NaOH}\;}{\mathrm{Sample}\;\mathrm{Weight}\;(\mathrm{mg})}\times 100\%,$$

(12) $$\mathrm{Galacturonic}\;\mathrm{Acid}\;\mathrm{Content}\;\left(\%\right)=\frac{176\;\times \;0.1z\;\times \;100}{\mathrm{Sample}\;\mathrm{Weight}\;(\mathrm{mg})}+\frac{176\;\times \;0.1y\;\times \;100}{\mathrm{Sample}\;\mathrm{Weight}\;(\mathrm{mg})},$$

where z is the volume (titer) of NaOH from the equivalent weight determination, y is the volume (titer) of NaOH from the methoxyl content determination, 31 is molecular weight of methoxyl in the form of CH_3_O, and 176 is the molecular weight of galacturonate.

## 5. Conclusions

This study used three independent variables which were found to affect the extraction of pectin from cocoa pod husk with microwave irradiation, namely, extraction time, microwave power, and solvent concentration. The effect of the three independent variables was analyzed on yield, temperature, moisture content, ash content, equivalent weight, methoxyl content, and galacturonic acid content. The results of the statistical test showed that only the model equations for temperature, moisture content, and ash content were suitable for predicting the response. The independent variables that significantly affected the three parameters were extraction time and microwave power. The overall best experimental conditions to produce the optimum parameter values were 30 min extraction time, 450 W microwave power, and 10% solvent concentration, resulting in a yield of 21.1%, temperature of 104 °C, moisture content of 6.00%, ash content of 8.00%, weight equivalent of 602.40%, methoxyl content of 6.07%, and galacturonic acid content of 72.86%.

## Figures and Tables

**Table 1:** Standard deviation and error of model equation.

Parameter	Standard Deviation	Error
Yield	0.254771	0.060743
Temperature	0.061314	0.063078
Water content	0.713623	0.116708
Ash content	0.820245	0.133777
Equivalent weight	2654.354	0.085203
Methoxyl content	0.008933	0.076808
Galacturonic acid content	1.408937	0.073445

**Table 2:** ANOVA results.

Parameter	F_count_	F_table_	SS (Sum of Squares) Regression	SS Total	*R^2^*
Yield	10.18	3.24	198.466	202.660	97.93%
Temperature	0.42		13.8218	14.7844	93.49%
Water content	0.15		60.0910	66.2000	90.77%
Ash content	0.83		72.8932	79.0000	92.27%
Equivalent weight	40.45		1306109	1337246	97.67%
Methoxyl content	19.24		3.94173	3.98390	98.94%
Galacturonic acid content	102.30		1682.00	1701.06	98.88%

**Table 3:** Lack of fit test results.

Parameter	*p*-Value for Lack of Fit
Yield	0.012
Temperature	0.817
Water content	0.973
Ash content	0.577
Equivalent weight	0.000
Methoxyl content	0.003
Galacturonic acid content	0.000

**Table 4:** Individual regression coefficient test results.

Parameter	*p*-Value		
	Extraction Time	Microwave Power	Solvent Concentration
Temperature	0.000	0.000	0.155
Water content	0.000	0.000	1.000
Ash content	0.001	0.000	1.000

**Table 5:** Simultaneous regression coefficient test results.

Parameter	*p*-Value	
	Linear Regression	Square Regression
Temperature	0.000	0.336
Water content	0.000	0.010
Ash content	0.000	0.001

**Table 6:** Normal distribution assumption test results.

Parameter	KS_count_
Temperature	0.150
Water content	0.255
Ash content	0.247

**Table 7:** Experimental design of pectin extraction.

No.	Extraction Time (Minutes)	Microwave Power (W)	Solvent Concentration (%)
1	20	180	2.5
2	30	180	2.5
3	20	450	2.5
4	30	450	2.5
5	20	180	10
6	30	180	10
7	20	450	10
8	30	450	10
9	20	300	5
10	30	300	5
11	25	180	5
12	25	450	5
13	25	300	2.5
14	25	300	10
15	25	300	5
16	25	300	5
17	25	300	5
18	25	300	5
19	25	300	5
20	25	300	5

## Data Availability

Publicly available datasets were analyzed in this study. These data can be found at http://repositori.usu.ac.id/handle/123456789/25569 (accessed on 5 February 2022).
